# Task-Driven Activity Reduces the Cortical Activity Space of the Brain: Experiment and Whole-Brain Modeling

**DOI:** 10.1371/journal.pcbi.1004445

**Published:** 2015-08-28

**Authors:** Adrián Ponce-Alvarez, Biyu J. He, Patric Hagmann, Gustavo Deco

**Affiliations:** 1 Center for Brain and Cognition, Computational Neuroscience Group, Department of Information and Communication Technologies, Universitat Pompeu Fabra, Barcelona, Spain; 2 National Institute of Neurological Disorders and Stroke, National Institutes of Health, Bethesda, Maryland, United States of America; 3 Department of Radiology, Lausanne University Hospital and University of Lausanne (CHUV-UNIL), Lausanne, Switzerland; 4 Signal Processing Lab 5, Ecole Polytechnique Fédérale de Lausanne (EPFL), Lausanne, Switzerland; 5 Institució Catalana de la Recerca i Estudis Avançats (ICREA), Universitat Pompeu Fabra, Barcelona, Spain; Université Paris Descartes, Centre National de la Recherche Scientifique, FRANCE

## Abstract

How a stimulus or a task alters the spontaneous dynamics of the brain remains a fundamental open question in neuroscience. One of the most robust hallmarks of task/stimulus-driven brain dynamics is the decrease of variability with respect to the spontaneous level, an effect seen across multiple experimental conditions and in brain signals observed at different spatiotemporal scales. Recently, it was observed that the trial-to-trial variability and temporal variance of functional magnetic resonance imaging (fMRI) signals decrease in the task-driven activity. Here we examined the dynamics of a large-scale model of the human cortex to provide a mechanistic understanding of these observations. The model allows computing the statistics of synaptic activity in the spontaneous condition and in putative tasks determined by external inputs to a given subset of brain regions. We demonstrated that external inputs decrease the variance, increase the covariances, and decrease the autocovariance of synaptic activity as a consequence of single node and large-scale network dynamics. Altogether, these changes in network statistics imply a reduction of entropy, meaning that the spontaneous synaptic activity outlines a larger multidimensional activity space than does the task-driven activity. We tested this model’s prediction on fMRI signals from healthy humans acquired during rest and task conditions and found a significant decrease of entropy in the stimulus-driven activity. Altogether, our study proposes a mechanism for increasing the information capacity of brain networks by enlarging the volume of possible activity configurations at rest and reliably settling into a confined stimulus-driven state to allow better transmission of stimulus-related information.

## Author Summary

Task- or stimulus-related changes of brain dynamics have been the subject of intense investigation during the last years. One of the most robust hallmarks of task/stimulus-driven brain dynamics, as measured using diverse recording techniques, is the decrease of variability with respect to the spontaneous level. This has led several researchers to focus on the second-order statistics of evoked activity and to study their functional consequences for information processing. In particular, it was observed that the trial-to-trial variability (related to variable responses to an identical stimulus from one presentation to the next) and the temporal variance of functional magnetic resonance imaging (fMRI) signals decrease in the task-driven activity. Here, we built a computational model of the whole brain to understand how local and large-scale brain dynamics contribute to these effects. The model allowed us to derive equations for the network statistics of both spontaneous and evoked activity. We observed that, as a consequence of single node and network dynamics, stimulus input impacts network statistics in such a way that the entropy of the stimulus-driven activity is lower than that during spontaneous activity. We confirmed this model prediction using empirical fMRI data and we further discuss its functional implications.

## Introduction

How spontaneous brain dynamics are altered under stimulation or task conditions remains an important open question in neuroscience. Empirically, one of the most robust hallmarks of task-driven brain activity is the decrease of variability following an external stimulus input, a phenomenon observed across a variety of species, cortical areas, tasks, stimulus and attentional conditions, and using brain signals observed across multiple spatiotemporal scales including neuronal membrane potentials, neuronal firing rates, field potentials and functional magnetic resonance imaging (fMRI) signals [1–5]. A recent fMRI study showed that trial-by-trial variability of BOLD signals decreases following stimulus onset in a visual detection task and that the magnitude of variability reduction was correlated with the magnitude of trial-averaged response [3]. Moreover, the temporal variance of BOLD signals is significantly smaller during the same task as compared with the resting condition [6]—an effect that has also been reported in brain field potentials, neuronal membrane potentials, and neuronal spiking activity [7–9]. This suggests that the multidimensional space outlined by cortical activity is reduced following the stimulus onset [10].

Yet, a detailed mechanistic explanation of these effects is still lacking. In the present work we aimed to model the empirical observations of the fMRI study of [3], by studying the effect of external inputs on the first- and second- order statistics of a large-scale model of the brain [11]. This model is composed of *N* local E-I nodes, with one excitatory and one inhibitory neural subpopulations, representing *N* brain regions that are interconnected through an empirical large-scale connectivity matrix obtained using diffusion imaging data of healthy human subjects [12]. The dynamics of each of the E-I nodes follows the mean field equations derived by [13] and the excitatory firing rate is clamped around 3 Hz by adjusting the connection weight from the I population to the E population, a procedure known as Feedback Inhibition Control (FIC) [11]. This large-scale model has been shown to provide an efficient description of resting-state fMRI functional connectivity together with realistic stimulus-evoked activity [11]. Here we assumed that different tasks can be modeled by sets of inputs that co-activate different brain regions. Furthermore, we focused on synaptic activity, since it has been shown that BOLD signals relate to local field potentials (LFPs) more closely than to neuronal firing rates [14–17].

Using this model we observed that, as a consequence of single node and network dynamics, the application of an external input impacts the network statistics, so that the entropy of the stimulus-evoked activity is lower than that during spontaneous activity. We confirmed this model prediction using empirical fMRI data and further discussed its functional implications.

## Results

In this work, we examined how external stimulation impacts the statistics of both single E-I nodes and a large-scale model composed of interconnected E-I nodes. A priori, the nonlinear and stochastic nature of the dynamical equations hinders analytical progress, and the calculation of the network’s statistics relies on numerical simulations of the stochastic differential equations (SDEs), which are time consuming and subject to sampling issues. But, in the case of weak noise, one can linearize the stochastic fluctuations and derive deterministic differential equations for the network’s statistics. This so-called *linear noise approximation* is described in the Methods section. In the following, unless otherwise specified, we used this method to approximate the network’s covariances, autocovariances, and power spectral densities both in the spontaneous condition and when an external input is applied to the network.

### Response of an isolated node

**Fig 1:**
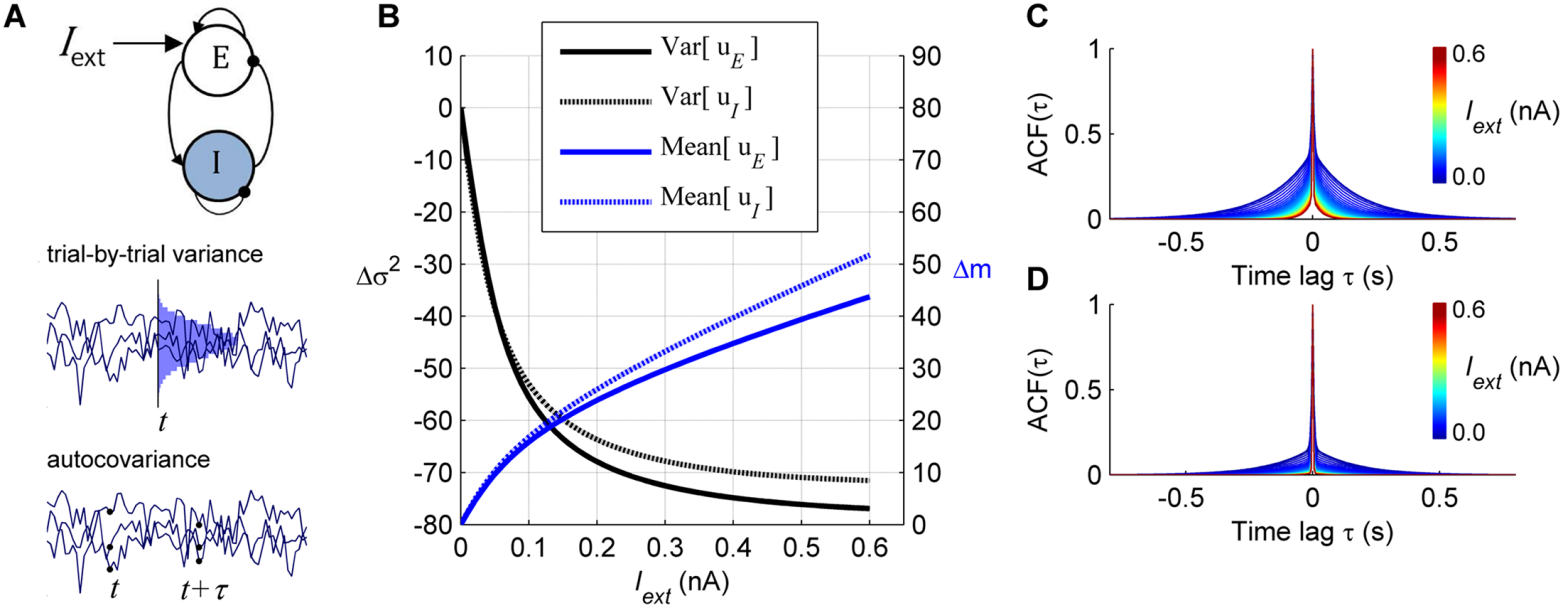
Response of a single local node. **A:** *Top*: Architecture of a local node composed of one excitatory (E) and one inhibitory (I) neural population. In the stimulated condition an external input *I*_ext_ is applied to the E population. *Middle*: The trial-by-trial variability describes the variance of synaptic activity at time *t* across stochastic realizations, i.e. simulation trials. Here, three trials are shown (the distribution of synaptic activity at time *t* is shown in *blue*. In the stationary regime, the trial-by-trial variance is independent of time. *Bottom*: The temporal variability describes the covariance of the synaptic activity with itself at pairs of time points, *t* and *t+τ*, averaged across stochastic realizations, i.e. simulation trials. Here, three trials are shown. In the stationary regime, the temporal variance is a function of *τ* only. **B:** Application of an external input increases the mean in both E and I populations (*blue*) while it reduces the synaptic trial-by-trial variance of both populations with respect to the spontaneous state (*black*). The amount of change of the variance was given by Δσ^2^(*I*_ext_) = 100x[σ^2^(*I*_ext_)–σ^2^(0)]/σ^2^(0). **C–D:** The application of the external input reduces the autocorrelation ACF(τ) of the excitatory population for the E population (C) and the I population (D). The colors indicate the intensity of the applied external input *I*_ext_.

We first evaluated the variability of the synaptic activity of single E-I nodes (Fig 1A). We calculated two types of variability: *i*) the variance across stochastic realizations of synaptic activity (trial-by-trial variance), noted *σ* ^2^, and *ii*) the autocovariance (temporal variance) of synaptic activity, defined as the covariance of the synaptic activity with itself at pairs of time points and noted *F*_*u*_(*t+τ*,*t*). Explicitly these statistics are given by:

$$\sigma^{2}=\text{Var}\left[u\right]=\langle {\left[u(t)-\langle u(t)\rangle \right]}^{2}\rangle ,$$ (1)

$$F_{u}\left(t+\tau ,t\right)=\langle \left[u(t+\tau )-\langle u(t+\tau )\rangle \right].\left[u(t)-\langle u(t)\rangle \right]\rangle ,$$ (2)

where *u* is the synaptic activity and the angle brackets <.> denote the average over stochastic model realizations (i.e. the average over simulated trials). The autocovariance measures the strength of the influence of the past dynamics of the system on its future dynamics [its normalized version, *F*_*u*_(*t+τ*,*t*)/*F*_*u*_(*t*,*t*), which is insensitive to the absolute amount of fluctuation, is the autocorrelation function (ACF)]. The equations governing these statistics can be analytically calculated by assuming that the noise is sufficiently weak to allow for a linearized treatment of the fluctuations or *linear noise approximation* (see Methods).

We examined how the application of an external stimulus *I*_*ext*_ to the E population (Fig 1A) changes the variability of synaptic activity. The stationary trial-by-trial variance of the synaptic activity under external input was compared to its stationary spontaneous level (*I*_*ext*_ = 0), and the relative change was quantified by:

$$\Delta \sigma^{2}(I_{ext})=100\times \left[\frac{\sigma^{2}(I_{ext})}{\sigma^{2}(0)}-1\right].$$ (3)

The relative change of the mean synaptic activity (Δm) was also computed:

$$\Delta \text{m}(I_{ext})=100\times \left[\frac{\text{m}(I_{ext})}{\text{m}(0)}-1\right].$$ (4)

Fig 1B shows that an external input monotonically reduces the trial-by-trial variance of the synaptic activity of both E and I populations, and increases the mean synaptic activity for both populations. In S1 Text we explicitly solved the equations for the variance and showed that the decrease of synaptic activity’s variance in response to an external input is determined by nonlinearities and connectivity parameters (see also S1 Fig).

Moreover, the external input reduces the spread of the autocorrelation function (ACF) of the synaptic activity of both E and I populations (Fig 1C and 1D). In conclusion, application of an external input attenuates the trial-by-trial fluctuations and shortens the temporal memory of the synaptic activity of an E-I local node.

### Response of the large-scale network

**Fig 2:**
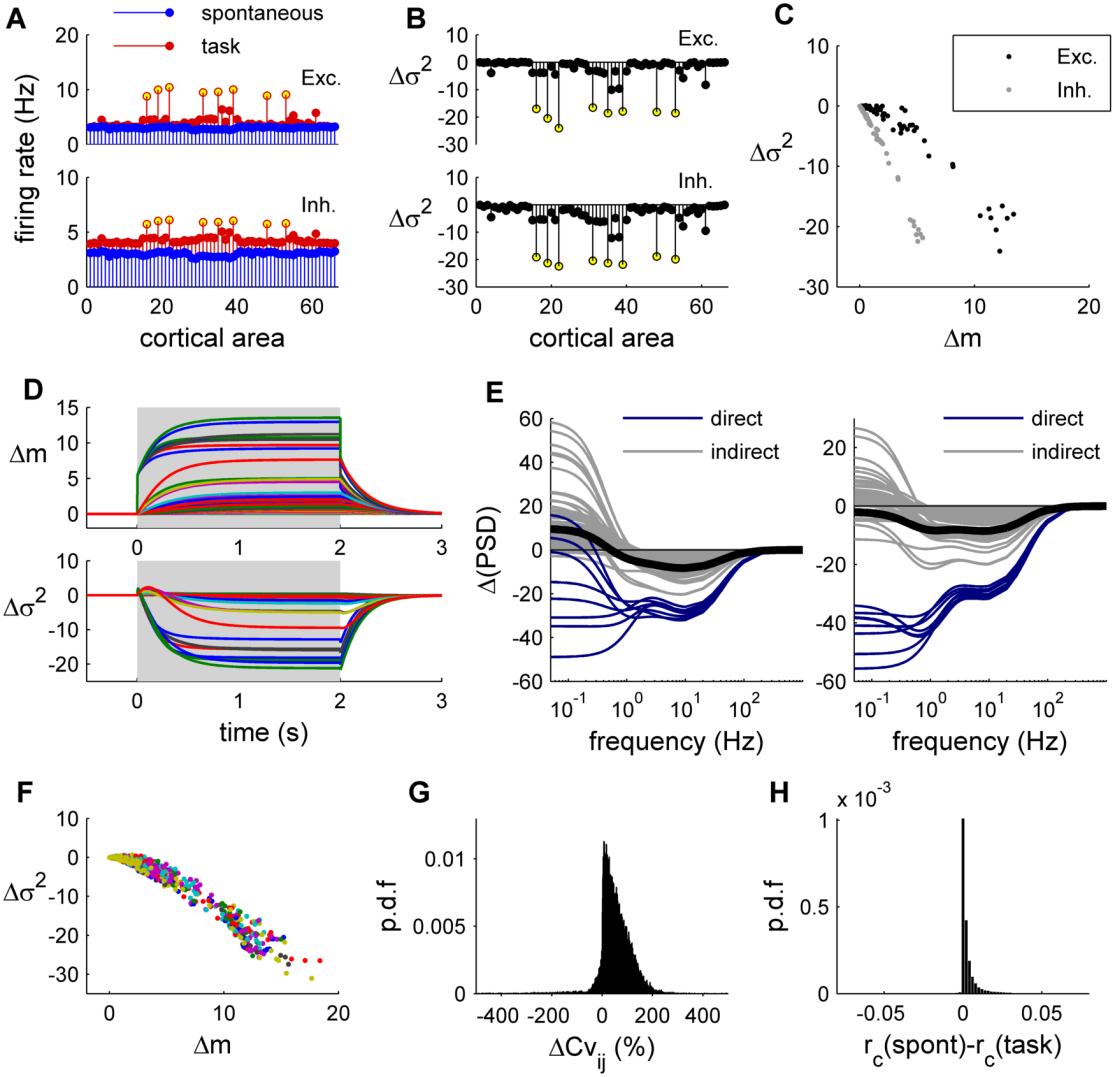
Response of the whole-brain network. **A:** Spontaneous (*blue*) and evoked (*red*) activity of the network for the excitatory populations (*top*) and the inhibitory populations (*bottom*). The yellow dots indicate the eight brain regions receiving the external input, which are: r/lLOCC, r/lMT, r/lPCAL, r/lST. **B:** Trial-by-trial variance change (Δσ^2^) with respect to the spontaneous condition for the excitatory populations (*top*) and the inhibitory populations (*bottom*). Yellow dots indicate the eight brain regions receiving the external input. **C:** Relation between the amount of mean synaptic change (Δm) and the amount of variance change (Δσ^2^) for the excitatory populations (*black*) and the inhibitory populations (*gray*). **D:** Temporal dynamics of Δm (*top*) and Δσ^2^ (*bottom*). Each line traces the time evolution of Δm (or Δσ^2^) of each brain region. The shaded area indicates the application of the stimulus. **E:** The stimulus-induced relative change of the power spectral density (ΔPSD) was computed for the excitatory populations (*left*) and the inhibitory populations (*right*). *Blue*: brain regions receiving a direct external input; *gray*: brain regions receiving an indirect external input; *black*: average across brain regions. **F:** The relation between Δm and Δσ^2^ is shown for 20 random stimulations constructed by randomly selecting eight nodes to which and external input is imposed (each color represents a given stimulation). **G:** Probability density function (p.d.f) of the relative change of the covariances between excitatory nodes. For each pair of nodes (*i*,*j*) we calculate the relative difference between the spontaneous covariance and the covariance evoked in the 20 random stimulations. **H:** Change of the correlations between excitatory nodes. For each pair of nodes (*i*,*j*) we calculate the difference between the spontaneous correlation coefficient and the correlation coefficient evoked in the 20 random stimulations.

We next evaluated the first- and second-order statistics of task-driven activity in a large-scale network composed of local E-I nodes interconnected through empirically derived anatomical connectivity (see Methods; see also [11]). The model has a single free parameter *G* that determines the strength of connectivity, called global coupling parameter (see Methods), which in the following is fixed to *G* = 2.15, this value falls in the range of *G* values (between 1–4.45) for which the model fits closely to the resting-state functional connectivity of fMRI data [11]. Given the previous results for an isolated node, we predict that external inputs to local nodes propagate through the dynamical system, reducing the trial-by-trial variance of other nodes in the network via direct or indirect pathways. Fig 2A shows the response of the large-scale network when eight brain regions receive an external input (equal to *I*_*ext*_ = 0.02 nA). To simulate the results of [3], in which human subjects performed a visual detection task, the selected brain regions receiving external inputs are related to visual processing. Two observations can be made: First, as expected from the response of isolated nodes, trial-by-trial variance reduces under simulated task condition for nodes directly receiving external inputs (Fig 2B). Second, consistent with the above prediction, many nodes not directly receiving external inputs also exhibit trial-by-trial variance reduction upon external stimulation to (other nodes in) the network. Notably, the change of trial-by-trial variance with respect to the spontaneous activity (Δσ^2^) is negative for all nodes (Fig 2B) and Δσ^2^ is negatively related to the change of synaptic activity Δm (Fig 2C). This negative relation is consistent with the empirically observed negative correlation between the magnitude of variability reduction and the amplitude of evoked response in fMRI signals [3]. This relation is expected for a large variety of connectivity matrices, since it arises from the propagation of the stimulus to nodes separated by direct and indirect links. However, using synthetic connectivities with different levels of clustering, we found that the relation holds for connectivity matrices with low or intermediate clustering, as it is the case of human connectomes, but it breaks for excessively clustered connectivity matrices for which recurrent connections highly dominate (see S2 Fig).

The temporal dynamics of the model (Fig 2D) show that, during the application of the stimulus, the mean synaptic activity increases, while its variance decreases, and, after a period of relaxation of ~1–2 s, the system settles into a stable stimulus-evoked state. In the stationary spontaneous and stimulus-induced states, the power spectral density (PSD) of fluctuations of the system in the presence of stochastic perturbations can be calculated using the linear approximation (see Methods, Eq 26). The change of variance in the frequency domain is given by the relative change of the power spectral density (ΔPSD) in the task-driven synaptic activity with respect to the spontaneous condition, defined as:

$$\Delta \text{PSD}=100\times \left[\frac{{\text{PSD}}_{task}}{{\text{PSD}}_{spont}}-1\right].$$ (5)

Interestingly, the effect of imposing an external input is different for different frequencies and, as a result of network interactions, the PSDs of the brain regions are differently affected by the external input (Fig 2E). For both excitatory and inhibitory units, most of the brain regions directly receiving the external input showed reduced power in frequencies lower than 100 Hz, with a maximal reduction at ~9Hz (9.65 Hz for excitatory units; 7.42 Hz for inhibitory units), but those not directly receiving external input showed increased power in frequencies below 0.9 Hz and decreased power in frequencies between 0.9 and 100 Hz, with a maximal reduction at ~9Hz (9.35 Hz for excitatory units; 8.42 Hz for inhibitory units). These results are consistent with empirical electrophysiological findings of prominent desynchronization in alpha/beta frequency ranges during task performance [18] and human ECoG observations of decreased power in <1 Hz range only in task-relevant brain regions [7].

To show that the above results are not specific to the particular hypothetical “visual” task, we produced a large set of hypothetical tasks, by imposing an external input (equal to 0.02 nA) to the excitatory population of 8 randomly selected brain regions. The negative relation between Δσ^2^ and Δm was found for all tested stimuli (Fig 2F). Interestingly, while the external stimulus highly impacts the covariances with respect to the spontaneous case (Fig 2G), with a tendency to increase them, it only slightly changes the correlations between nodes (Fig 2H). This indicates that functional connectivity amongst nodes, classically measured using correlation matrices, is not dramatically changed by imposing an external stimulus.

**Fig 3:**
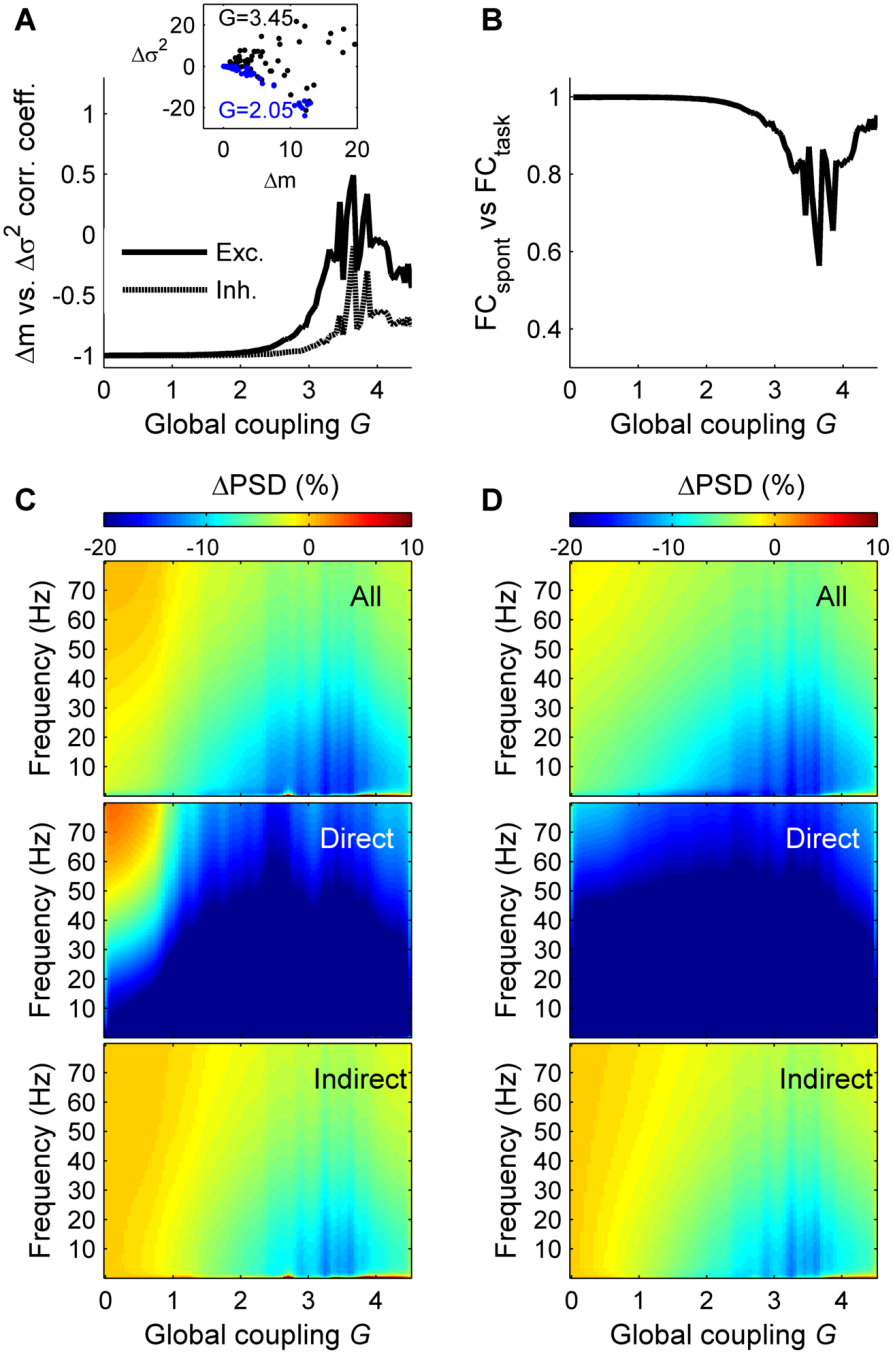
Network response vs. global coupling. **A:** Correlation coefficient between the amount of mean synaptic change (Δm) and the amount of variance change (Δσ^2^) as a function of *G*, for the excitatory populations (solid line) and for the inhibitory populations (dashed line). The inset shows the relation between Δm and Δσ^2^ for *G* = 2.05 (*blue*) and *G* = 3.45 (*black*) for the excitatory populations. **B:** Similarity (correlation coefficient) between the spontaneous and the task-evoked functional connectivity matrices as a function of the global coupling (*G*). **C:** Relative change of the power spectral density (ΔPSD) in the stimulated condition compared to the spontaneous condition, as a function of *G*, for the excitatory populations. *Top*: ΔPSD averaged across the excitatory populations of all brain areas. *Middle*: ΔPSD averaged across the excitatory populations of the brain areas directly receiving the external input. *Bottom*: ΔPSD averaged across the excitatory populations of the brain areas indirectly receiving the external input. **D:** same as C for the inhibitory populations. In A–D the brain regions receiving the external input are: r/lLOCC, r/lMT, r/lPCAL, r/lST.

We next allowed the global parameter *G* to vary and observed that the above results are qualitatively the same for a large parameter space (within *G* = 1 and 3) (Fig 3), namely that Δσ^2^ and Δm are negatively related (Fig 3A), that the task-driven functional connectivity is very similar to the spontaneous functional connectivity (Fig 3B), and that the input prominently reduces the power of frequency fluctuations lower than 40 Hz (Fig 3C and 3D). Within this parameter range the model captures both the observed behavior of the stimulus-driven activity and the resting functional connectivity (as shown previously in [11]). In contrast, for *G*>3, the model correctly predicts the resting functional connectivity, but the behavior of the stimulus-driven activity is not consistent with the empirical observations.

**Fig 4:**
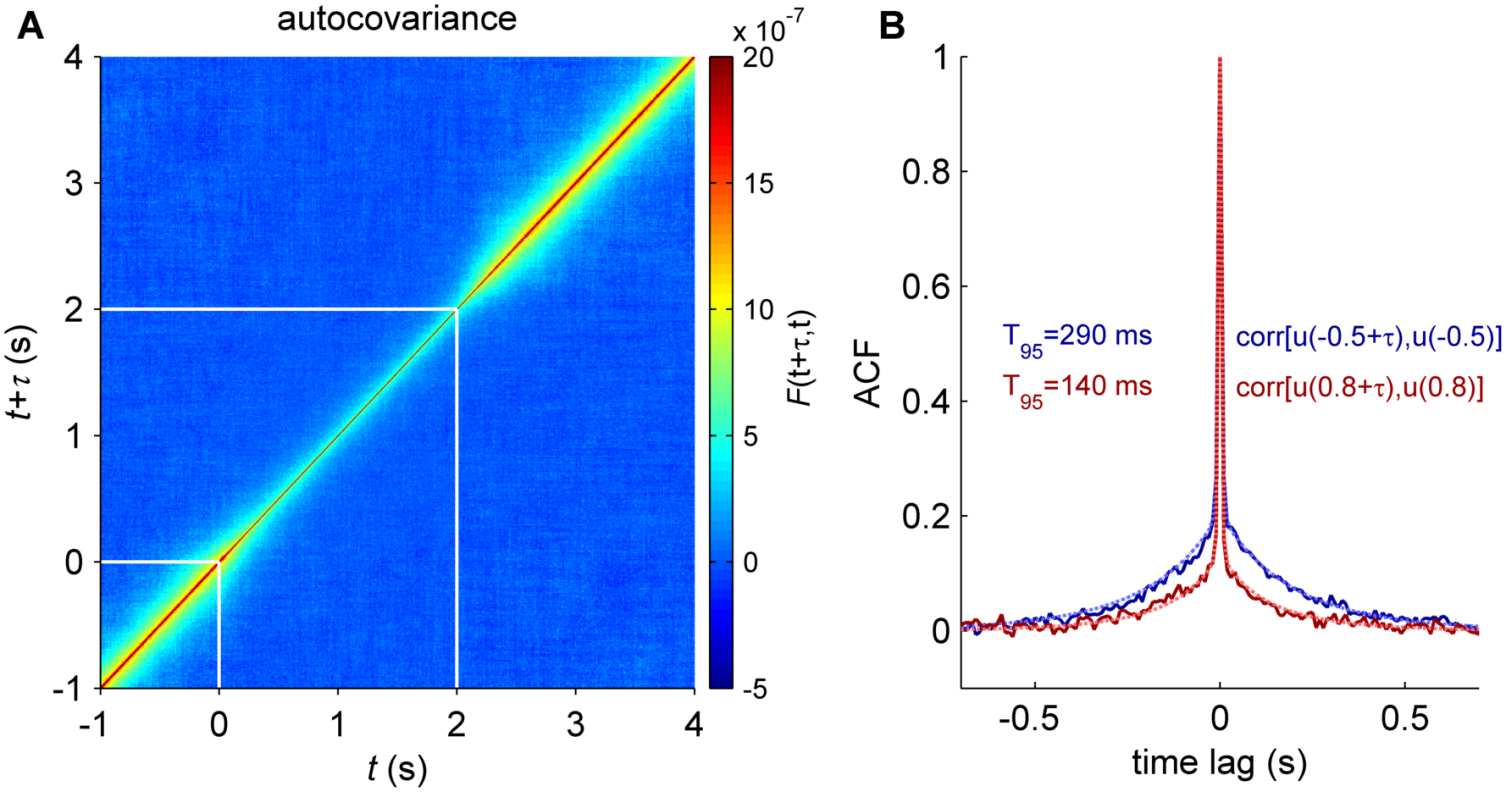
Time correlations. **A:** The autocovariance function was calculated for each time step time *t* and averaged across excitatory populations of all brain regions. The stimulus was presented at *t* = 0 at last for 2 s (white lines). **B:** The averaged autocorrelation function (ACF) of excitatory synaptic activity at two different times, *t* = 0.5 s (spontaneous, *blue*) and *t* = 0.8 s (stimulus period, *red*). Solid lines are results from simulations and the dotted *blue* and *red* lines corresponded to the linear approximation in the stationary regime for the spontaneous and stimulus conditions, respectively. T_95_ represents the characteristic time-scale: the time lag at which the ACF is equal to 0.05. In this simulation the brain regions receiving the external input are: r/lLOCC, r/lMT, r/lPCAL, r/lST.

We next calculated the dynamic change of the temporal variance (autocovariance) of the synaptic activity when an external input is applied to the large-scale model. The external input was applied at time *t* = 0 and lasted for 2 s. We used direct stochastic simulations of the network to estimate the time evolution of the autocovariance (Fig 4A). During the application of the external input, the temporal correlation length is reduced. To quantify this effect we calculated the characteristic time scale of the ACF, noted T_95_, given by the time lag at which its value is equal to 0.05 (i.e. 95% percent of correlation decay). T_95_ was calculated using the linear approximation (Eqs (21–24)) in stationary spontaneous and stimulus conditions (Fig 4B). We found that temporal correlations lasted more than twice as long in the spontaneous state than in the task state (for the excitatory synaptic activity: T_95_ = 290 ms vs. T_95_ = 140 ms; for the inhibitory synaptic activity: T_95_ = 170 ms vs. T_95_ = 30 ms). Hence, the temporal memory of the synaptic activity of the large-scale model is shortened after the stimulus onset.

### Simulated BOLD dynamics

**Fig 5:**
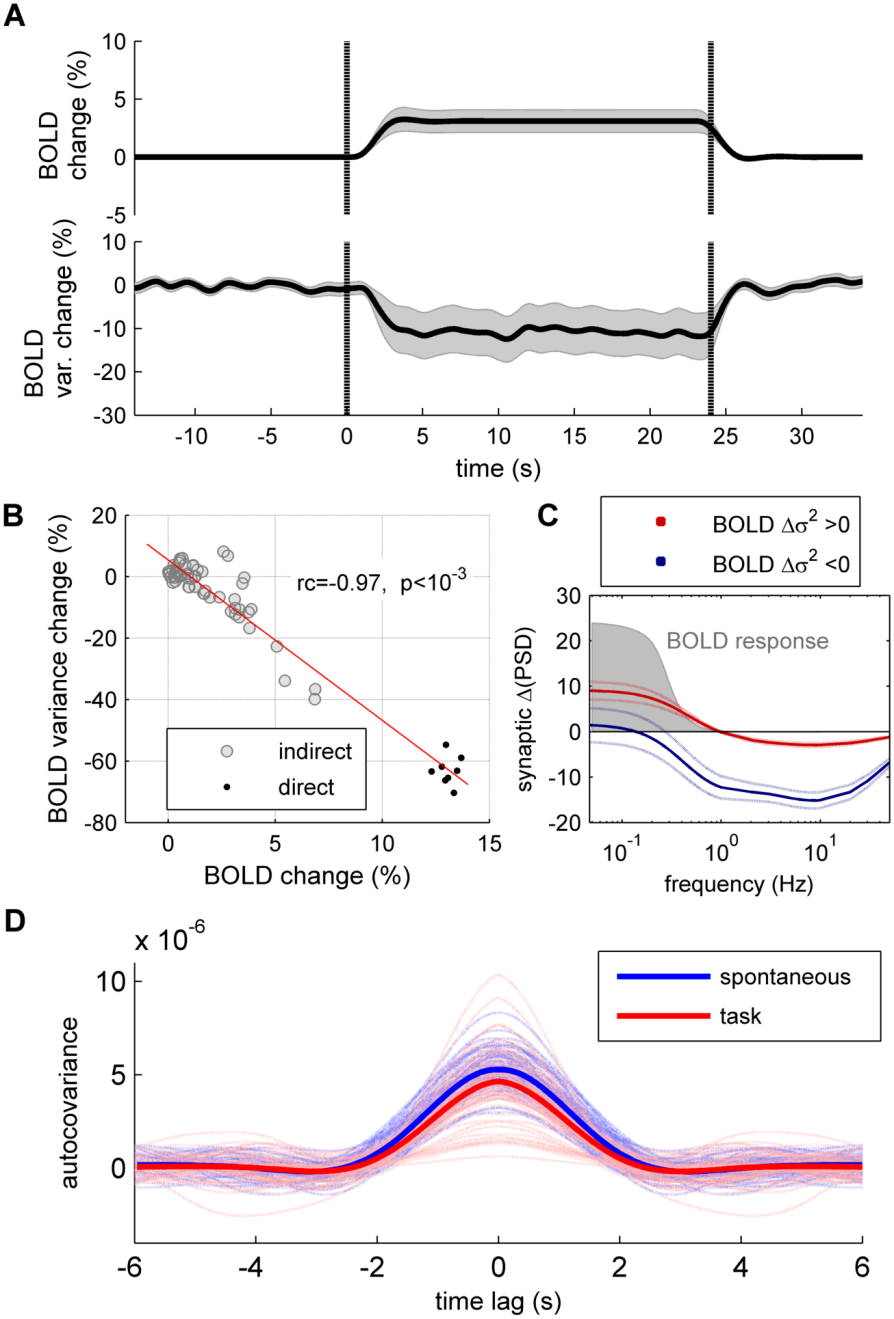
Spontaneous and evoked dynamics of simulated BOLD signals. **A:** The BOLD activity was simulated in response to a stimulus application for 800 trials. *Top*: evolution of the trial-averaged BOLD activity relative change, with respect to the spontaneous period (before the stimulus onset), averaged across brain regions. *Bottom*: BOLD trial-by-trial variance relative change, with respect to the spontaneous period, averaged across brain regions. Shaded areas represent 95% confidence intervals. Vertical dotted lines indicate the onset and offset of the stimulus. **B:** Relation between the amount of mean BOLD change and the amount of variance change of BOLD signals, averaged over the period between *t* = 5s and *t* = 23s, for the brain regions receiving directly (*black*) and indirectly (*gray*) the external input. **C:** Averaged change in power spectral density of the summed E-I synaptic activity (ΔPSD) separately for those brain regions that show an increase (*red*) or a decrease (*blue*) of variance in the simulated BOLD signal. The shaded area indicates the response function of the BOLD model, given by the amplitude of the BOLD signal in response to cosine inputs of different frequencies, i.e. zi = cos(ωt). **D:** Autocovariance of the BOLD signals of individual brain regions in the spontaneous condition (*light blue*) and in the stimulated condition (*light red*). The autocovariance averaged across brain regions is shown in *blue* for the spontaneous condition and in *red* for the stimulated condition. In A–C the brain regions receiving the external input are: r/lLOCC, r/lMT, r/lPCAL, r/lST.

Up to now we have focused on the dynamics of the synaptic activity. Because BOLD fMRI is widely used to study brain dynamics under both resting state and cognitive tasks, an important question pertains to whether the previous results apply to the dynamics of BOLD signals. To test this, we used a hemodynamic model to convert the total synaptic activity (the sum of excitatory and inhibitory synaptic activity) into BOLD activity. We used the Balloon-Windkessel model for the Hemodynamic response that describes the transduction of neural activity to BOLD changes, though non-linear dynamic equations of blood flow and deoxyhemoglobin content [19]. The model parameters were chosen as in [3]. Using this nonlinear model we found that an external stimulus input increases the trial-averaged BOLD activity, while reducing the averaged trial-by-trial variance of BOLD signals (Fig 5A), leading to a linear negative relation between the relative change of trial-averaged BOLD activity and the relative change of its trial-by-trial variance during the application of the external input (Fig 5B). However, the relative change of variance is positive for some of the brain regions (23 over 66). In the model, this is due to the low-pass filtering of the hemodynamic model, since the Balloon-Windkessel model acts as low-pass filter of the synaptic activity that passes frequencies under 1 Hz [20, 21]. As shown in Fig 2E, the stimulus-induced decrease of the synaptic variance is not negative for all brain regions for frequencies under 1 Hz. As a consequence, those brain regions for which the synaptic activity presents an elevation of the spectral power under 1Hz have a positive relative change of the variance of the BOLD activity (Fig 5C). The stimulus-induced reduction of the autocovariance (Fig 5D) is moderate for the same reason: the memory of the BOLD signal is highly dominated by the slow hemodynamic response.

### Entropy reduction and relative entropy

**Fig 6:**
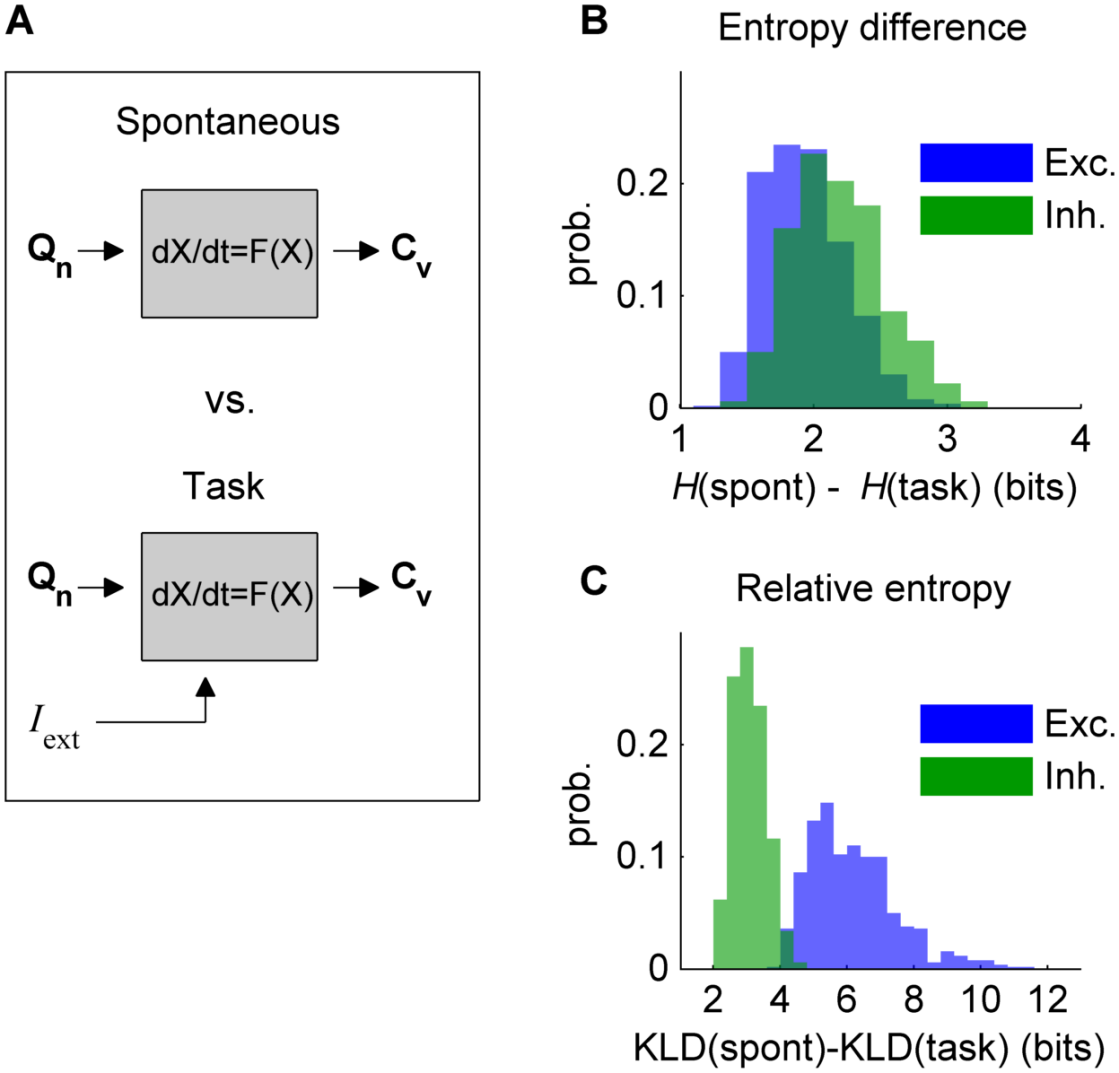
Entropy and relative entropy. **A:** The input noise (with covariance **Q**_**n**_) propagates through the dynamical system and generates an output covariance **C**_**v**_ between the system’s state variables (i.e. synaptic activity). The differential entropy of the input process (noise) and the output process (synaptic activity) is determined by the corresponding covariance of inputs and outputs. The differential entropy of the inputs and the one of the outputs of the large-scale model were compared in the spontaneous and task (stimulated) conditions. **B:** The differential entropy *H* of the large-scale model was calculated in both the spontaneous state and for many (500) random stimulations constructed by imposing an external input (equal to 0.02 nA) to the excitatory population of 8 randomly selected brain regions. The spontaneous entropy is 1–3 bits larger than the entropy in the task-evoked condition, for both excitatory (*blue*) and inhibitory (*green*) synaptic activity. **C:** The relative entropy was measured as the amount of entropy of the synaptic activity that cannot be accounted for by the noise inputs. It is given by the Kullback-Leibler divergence (KLD) between the input stochastic process and the observed synaptic activity. The relative entropy is larger in the spontaneous state than in the stimulated condition, for both excitatory (*blue*) and inhibitory (*green*) synaptic activity. In (B) and (C) the noise covariance **Q**_**n**_ is diagonal (i.e., intrinsic noise is uncorrelated).

We next investigated the functional implications of the change in network statistics induced by external inputs. To this end, we calculated the differential entropy *H* of the synaptic activity. The differential entropy is an extension of the Shannon entropy for a continuous random variable and it is related to the volume occupied by the continuous random variable. *H* can be easily calculated for a multivariate normal distribution, an assumption that is met in our case for the level of noise used in this study (S3 Fig). In such cases, *H* depends on the covariance matrix which can be calculated using the linear noise approximation (see Methods). We evaluated the differential entropy of the spontaneous activity and of the stimulus-driven activity for different model tasks determined by external inputs to a given subset of brain regions (Fig 6A). We found that external stimulation systematically reduces the entropy of the synaptic activity (Fig 6B).

We next asked how much entropy (or uncertainty) in the synaptic activity is explained by the intrinsic noise present at each node of the model. In other words, we asked how much uncertainty is produced by the dynamical system due to the intrinsic noise of each node propagating into the network. To answer this question we calculated the Kullback-Leibler divergence (KLD), also called relative entropy, between the distribution of intrinsic noise and the distribution of synaptic activity. Because the intrinsic noises are normally distributed with covariance **Q**_**n**_ and the distribution of synaptic activity is normally distributed (for weak noise) with covariance **C**_**v**_, the KLD can be calculated using Eq (32) (see Methods). We found that the relative entropy of the spontaneous synaptic activity is systematically higher than that of the stimulus-driven synaptic activity, indicating that in the spontaneous state the dynamical system adds more uncertainty to the intrinsic stochastic process than it does in the stimulated condition (Fig 6C).

Thus far we have considered that the intrinsic noise of each brain region is independent between nodes (i.e., **Q**_**n**_ is diagonal). However, it is reasonable to think that during a task and even at rest different brain regions share some noise, possibly due to shared sensory/proprioceptive background inputs. We thus calculated the entropy and the relative entropy in the case of non-diagonal noise covariance matrices. As for the diagonal case, we found that the stimulus-driven synaptic activity has lower differential entropy and lower relative entropy than the spontaneous activity (S4 Fig). Thus, external stimulation reduces the entropy of synaptic activity even in the presence of common noise.

### Entropy reduction of fMRI signals

**Fig 7:**
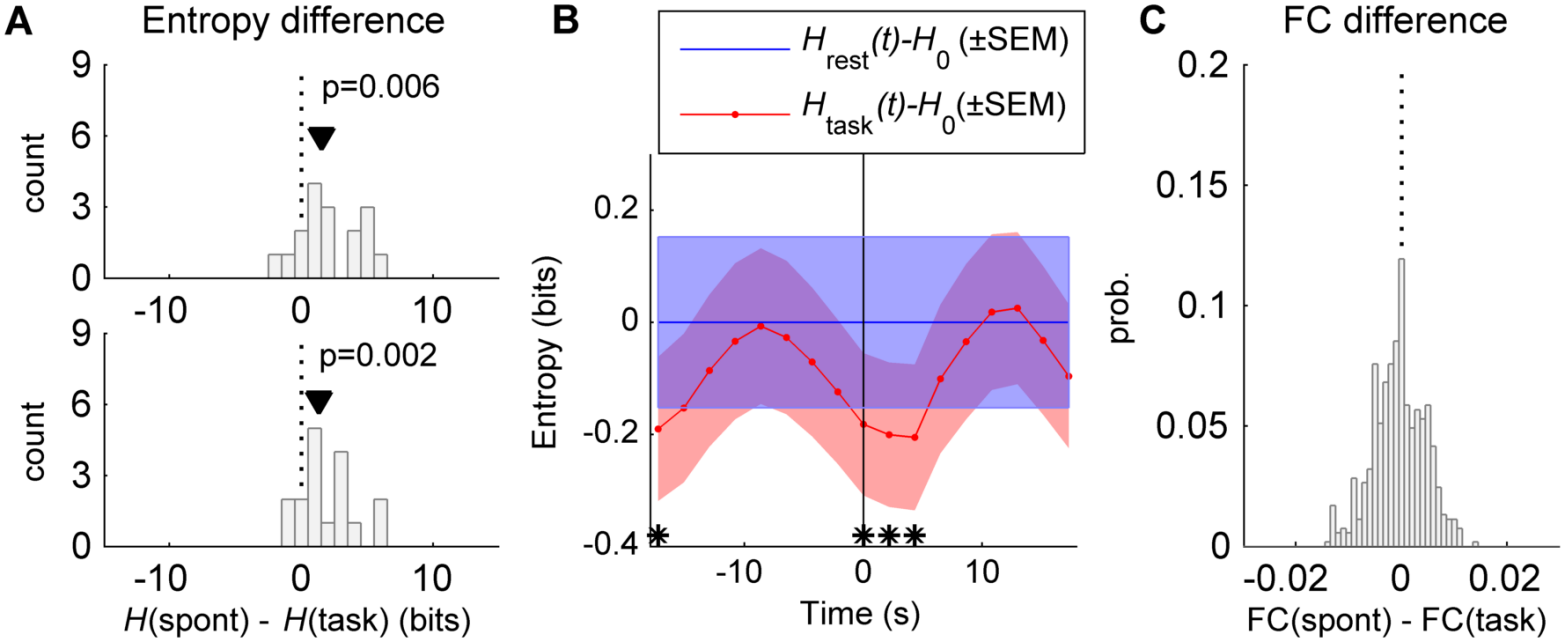
Entropy of fMRI signals. **A:** The differential entropy *H*(spont) of the resting fMRI BOLD activity was compared with the differential entropy *H*(task) of the task-driven fMRI BOLD activity. The differential entropy was estimated with two different methods: assuming that fMRI signals are represented by a multivariate Gaussian process (*top*) and using the Nilsson-Kleijn non-parametric estimator (*bottom*). The black triangle indicates the median of the entropy difference *H*(spont)–*H*(task) (p: p-value for the Wilcoxon signed-rank test for zero median). **B:** Peristimulus time resolved differential entropy. The differential entropy was calculated in windows of 5 frames, moved in steps of 1 frame, using Eq (30), in both rest [*H*_rest_(*t*)] and task [*H*_task_(*t*)] conditions and then averaged across subjects. Entropy values were referenced to *H*_0_, i.e. the entropy averaged across subjects and time windows of 5 frames in rest condition. For task activity the data was aligned to the stimulus onset (vertical line) and the entropy was averaged over stimulus presentations in the period between 17.3 s before the stimulus onset and 17.3 s after the stimulus onset; for rest activity, the entropy was averaged over equivalent periods. The *blue* and *red* shaded areas indicate the SEM of [*H*_task_(*t*)–*H*_0_] and [*H*_rest_(*t*)–*H*_0_], respectively. The stars indicate the windows during which *H*_task_(*t*) is significantly different than *H*_rest_(*t*) (p<0.01, paired *t*-test). **C:** distribution of the difference between the correlation coefficients of the functional connectivity (FC) of resting activity, averaged across subjects, and the FC of task activity, averaged across subjects.

We tested the model prediction of higher entropy in the spontaneous activity than in the task-driven activity using empirical data from [3]. The data consists of fMRI time-series from 33 ROIs, covering five cortical networks, as well as the hippocampus, thalamus and cerebellum, acquired in 17 healthy subjects (see Methods). Each subject completed 8 fMRI runs, each lasting ~7 min, including 4 runs in resting-state conditions and 4 runs in a visual detection task condition. Each task run contains 20 stimulus presentations that the subject was to detect by pressing a button as quickly as possible. The inter-stimulus interval ranged from 17.3–30.2 s. First, for each subject and condition, we concatenated the time-series of the different runs and estimated the entropy using two methods: i) by assuming normality and using Eq (30), and ii) by using the Nilsson-Kleijn non-parametric estimator (see Methods). Using both methods, we found that the differential entropy in the resting activity is significantly higher than that of the task-driven activity (p<0.01, Wilcoxon signed-rank test) (Fig 7A). Second, we performed a time-resolved analysis in which the differential entropy was calculated using sliding windows of 5 frames (10.8 s) shifted in steps of 1 frame (2.16 s). Using Eq (30), we computed the time course of the differential entropy, averaged across subjects, in the task condition, *H*_task_(*t*), and during rest, *H*_rest_(*t*) (Fig 7B). The entropy values were referenced to the rest entropy *H*_0_ averaged across subjects and across time windows, i.e. $H_{0}=\frac{1}{T}{\sum_{t=1}^{T}H}_{\text{rest}}(t)$, where *T* is the total number of time steps in a run. When the task data was aligned to the stimulus onset, we found that the differential entropy significantly decreases after the stimulus onset (p<0.01, paired *t*-test) and, after ~8 s, it recovers its resting level *H*_0_ (the significant difference at –17.3 s is due to the previous stimulus).

Interestingly, the resting-state functional connectivity and the task functional connectivity were very similar, with differences in correlation coefficients ranging between ±0.02 (Fig 7C), a feature that is captured by the model (see Fig 2H).

### Reduction of the cortical activity space

**Fig 8:**
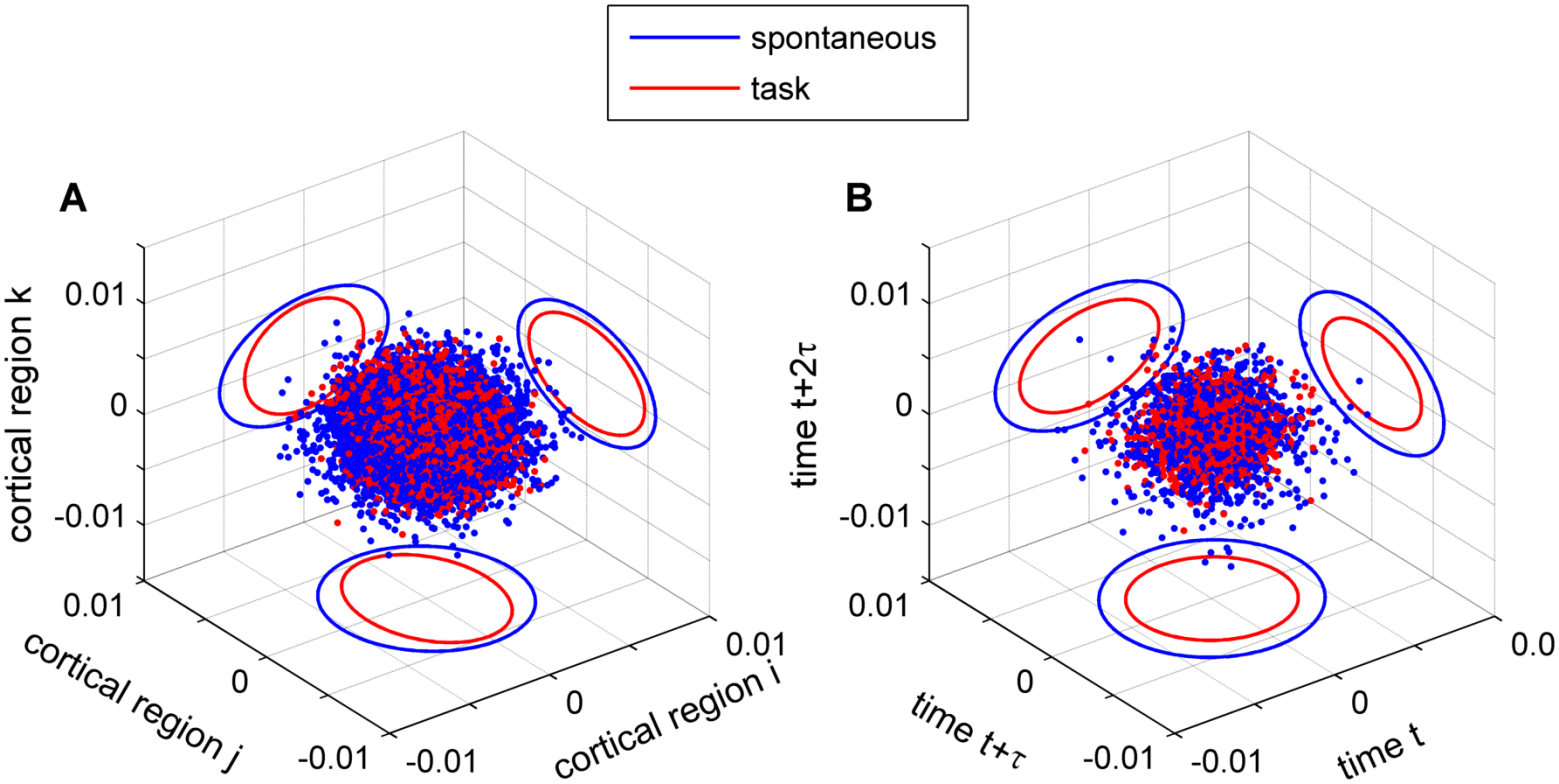
Reduction of the cortical activity space. **A:** The excitatory synaptic activity of three brain regions, in spontaneous (*blue*) and task (*red*) conditions, is plotted in a three-dimensional space: each dot represents the vector X_i,j,k_(t) = [x,y,z], where x = u_i_(t), y = u_j_(t), z = u_k_(t), and *i*, *j*, and *k* denotes three brain regions. For this particular model task eight brain regions receive an external input; here, region *i* receives a direct external input while regions *j* and *k* receive indirect inputs through large-scale connections. The ellipses represent the 95% confidence of the distribution projected onto two-dimensional planes. **B:** The excitatory synaptic activity of a single brain region *i*, in spontaneous (*blue*) and task (*red*) conditions, is plotted in a three-dimensional Poincaré map: each dot represents the vector X = [x,y,z], where x = u_i_(t), y = u_i_(t+τ), z = u_i_(t+2τ), and τ = 200ms. The ellipses represent the 95% confidence of the distribution projected onto two-dimensional planes.

The above results show that the task-driven synaptic activity has lower trial-by-trial variance, lower temporal variance, and lower entropy than the spontaneous synaptic activity. Altogether, this indicates that the space occupied by the synaptic activity is reduced when external inputs are impinging upon the network. To illustrate this effect, we represented the synaptic activity at a given time point or in a given trial as a point in the state space. Fig 8A shows the simulated synaptic activity of three brain regions in a three-dimensional space defined by the activity of these brain regions when no external input is applied (spontaneous condition) and when an external input is applied (task condition). The mean activity was removed for each brain region; thus, here, the activity represents deviations from the mean. In the spontaneous condition, the network explores a volume of the state space that is larger than the volume explored in the task condition. In the temporal domain, the space occupied by the synaptic activity is also reduced in the task condition compared to the spontaneous condition (Fig 8B). This is shown by plotting the synaptic activity of a given brain region *i* in the three-dimensional space, or Poincaré map, defined as the synaptic activity in three different time points *t*, *t*+τ, and *t*+2τ. The volume of the space in the Poincaré map outlined by the spontaneous synaptic activity is larger than that occupied by the task-driven synaptic activity.

## Discussion

We have shown that external stimulation to a large-scale brain model attenuates the synaptic fluctuations, increases the covariances, and reduces temporal memory in brain regions receiving the input directly or indirectly through the anatomical connectivity. Furthermore, we showed that the spontaneous activity has more entropy and more relative entropy than the task-driven activity. More entropy and more relative entropy means that the brain network produces a larger number of possible activity configurations that are not explained by the intrinsic noise. In other words, as shown in Fig 8 and in accordance with empirical observations, the multi-dimensional synaptic activity space is larger in the spontaneous state than under external inputs.

### Information capacity and transmission

Reducing the space occupied by the synaptic activity, as a consequence of reducing the trial-by-trial and the temporal variability and increasing covariances, has relevant implications for information processing. It has been shown that temporal variance and entropy of BOLD signals change with chronological age and that young adults who are also faster and more consistent performers exhibited significantly higher brain variability across tasks [22–24]. In addition, the reduction of trial-by-trial variability is highly predictive of better performance [4]. These observations are likely complementary. Indeed, in the view of Information Theory, the mutual information between the brain activity and a given stimulus can be decomposed as the difference between the entropy of the full set of response patterns for all stimuli (total entropy) and the entropy conditioned to one stimulus (evoked entropy). Thus, there are two ways of increasing the information carried by the brain activity: by increasing the total entropy or by decreasing the evoked entropy. There is growing evidence that the entropy at rest is an upper bound of the total entropy, since stimulus-evoked patterns reoccur during spontaneous activity [10, 25, 26]. In other words, more variability at rest is associated with a larger repertoire of potential brain states and greater information capacity [27] while the ability to reliably settle in a stimulus-evoked brain state allows better transmission of the information about the stimulus.

Information Theory provides quantification of the amount of potential information that is available given the distribution of brain activity. How the brain decodes this available information is a topic of active research. Classification of multivariate fMRI patterns has been used to decode different stimuli or behavioral conditions from the fMRI signals [28–30]. In this context, the reduction of trial-by-trial variability under task would improve the discriminability of the fMRI multivariate patterns, which in turn improves the decoding performance. Moreover, if multivariate patterns have to be estimated using short time windows, as is likely during dynamical task processing, reducing the temporal correlations of the fluctuations would improve the estimation of the patterns (since the reduction of the autocorrelation leads to an increase of the effective number of independent samples within the time window). It is possible that the brain uses similar coding schemes to efficiently represent the incoming sensory information and evolving mental states, although exactly how such decoding schemes are implemented by neural systems remains an open question.

### The link between synaptic activity and BOLD signals

In the present study we focused on the dynamics of the synaptic activity to model the empirical BOLD fMRI signals. Concentrating on the synaptic activity is justified since it has been shown that BOLD signals relate more closely to Local Field Potentials (LPF) rather than neuronal firing rates [14–17]). As in previous studies of large-scale models [11, 21, 31], we converted the synaptic activity into simulated BOLD signals via a non-linear hemodynamic model, known as the Balloon-Windkessel model [19]. We found prominent stimulus-induced decrease of BOLD variance and a negative correlation between the relative change of trial-averaged BOLD responses and the relative change of trial-by-trial BOLD variance, as reported empirically by [3]. This supports a previous conclusion [3] that the observed BOLD variability reduction is unlikely to be an effect of the nonlinearities in the hemodynamic response but rather is likely due to the underlying synaptic activity.

Nonetheless, for some brain regions the simulated BOLD activity has slightly more variance in the stimulus-driven activity than in the spontaneous activity (Fig 5B). Using a voxel-wise analysis across the whole brain on empirical fMRI data, it was found that whereas some voxels showed increased variance after stimulus onset, none of them were statistically significant after correction for multiple comparisons (Figure 4 in [3]). Thus, whether the observation of task-induced increase in trial-to-trial variance in selected regions in our model has physiological importance remains to be seen. As mentioned above, the increase of variance in the present model is due to the low-pass filtering of the hemodynamic model that suppresses the fluctuations frequencies above 1 Hz. Indeed, consistent with previous empirical observations using human ECoG recordings [7], the present model shows a prominent task-induced decrease of synaptic variance for frequencies >1Hz, but, for frequencies <1Hz, this is mostly evident in directly activated brain regions only. This suggests that the present dynamic mean field model might be too simple to reconcile these two features and should be extended to consistently reproduce the change of spectral power in both synaptic and BOLD activities. Another alternative is that the hemodynamic model needs to be refined to completely describe the neurovascular coupling between the BOLD signal and the synaptic activity at different frequencies. Indeed, experimental evidence shows that BOLD fluctuations correlate with broadband LFP signals and that the alpha (8–12 Hz), beta (18–30 Hz), and gamma (40–100 Hz) LFP bands were informative about the spontaneous BOLD signals from an individual brain area [32].

### Mechanism of variability reduction in the stimulus-evoked activity

The mechanism underlying stimulus-induced decrease of neural variability has been recently studied in theoretical works. Among the proposed mechanisms, spontaneous multi-stability has received much attention [5, 33, 34]. Under this scenario, the spontaneous activity of local neural networks with an underlying clustered connectivity is highly variable due to transitions through multiple spontaneous states. These transitions render the spontaneous activity more heterogeneous, but are suppressed when a stimulus stabilizes the network in a single evoked state and, as a result, the variability decreases in the stimulus-driven activity. This scenario naturally predicts an important feature of spontaneous activity, namely that the different spontaneous states are similar to the stimulus-evoked states [35, 36], a phenomenon reported in studies of neuronal membrane potentials and spiking activity at the microcircuit level [10, 25] and in resting-state fMRI studies at large-scale network level [26, 37–40]. By contrast, in the present study, the reduction of variability is due to single node synaptic dynamics (Fig 1) without the need of multi-stability originating from clustered connections. We showed that variance decrease results from nonlinearities and local E-I connectivity (see S1 Text). When the local nodes interact through long-range connections a pattern of stimulus-induced variance reduction is observed as a result of direct and indirect inputs—a phenomenon that is expected for a large variety of connectivities, as soon as large-scale recurrent connections do not strongly dominate (S2 Fig). The model for local nodes presented herein is a mean-field model that describes the mesoscopic dynamics of synaptic activity. This model can be extended by introducing multi-stability in the local dynamics, a direction that requires further investigation.

### Rest vs. task functional connectivity

In the present data and model the functional connectivity is only slightly changed between rest and task. Several studies have reported high similarity between resting and task-related functional connectivity [37–40]; however, other studies have demonstrated reorganization of functional networks during task performance [41–43]. Brain dynamics might be engaged into task activity through diverse mechanisms. Here we modeled task-driven activity by imposing sets of inputs that co-activate different brain regions. There are other possible models for task effects on the brain, such as neuromodulation-mediated changes of network parameters that modify the neural excitability, the synaptic efficacy, or the gating of inputs. How these mechanisms alter the statistics of task-driven activity in a large-scale model have only recently been examined and awaits further investigation [44–46].

### Time-varying inputs

Finally, we here focused on the effect of imposing a stationary input to the large-scale brain model. A natural extension of the present work would be to study the effect of time-varying (sinusoidal) inputs and compute the frequency-dependent response function of different network statistics. Considering an input of small amplitude would allow to linearize the response and to study the eventual resonances. Moreover, these resonances may be partly determined by transmission delays, given by the experimental distance matrix between the different brain regions, a scenario that is not consider in the present work.

### Conclusion

In conclusion, we have shown that the stimulus-driven shrinkage of cortical activity space can be understood as a property of mesoscopic dynamics embedded in large-scale brain networks, a property that has important implications for information processing.

## Methods

### Ethics statement

This research was conducted in agreement with the Code of Ethics of the World Medical Association (Declaration of Helsinki) and informed consent was obtained from all subjects before performing the study, in accordance with institutional guidelines. The study design was approved by the Human Studies Committee of Washington University in St. Louis and the local Ethics Committee of Lausanne University.

### Empirical fMRI data collection and analysis

Blood-oxygen-level dependent (BOLD) fMRI data (4x4x4 mm^3^ voxels, TE 25 ms, TR 2.16 s) were acquired in 17 normal right-handed young adults (9 females, age 18–27 years) using a 3T Siemens Allegra MR scanner. All subjects gave informed consent in accordance with guidelines set by the Human Studies Committee of Washington University in St. Louis. Each subject completed 8 fMRI runs, each 194 frames (~7 min) in duration. They consisted of two alternating run types. The first run type was a resting-state study in which a white crosshair was presented in the center of a black screen. Subjects were instructed to look at the crosshair, remain still, and to not fall asleep. The second run type was a task study in which the identical crosshair was presented, but now it occasionally changed from white to dark gray for a period of 250 ms, at times unpredictable to the subjects, with an inter-stimulus interval of 17.3–30.2 sec. The subjects were instructed to press a button with their right index finger as quickly as possible when they saw the crosshair dim. This data set has been previously used in [3, 6, 47]. Thirty-three regions of interest (ROIs) covering five cortical networks—the attention, default-mode, motor, saliency and visual networks, as well as the hippocampus, thalamus and cerebellum were defined based on previous task-related functional neuroimaging studies. The preprocessing of the fMRI data and definition of ROIs are described in detail in [3].

### Large-scale cortical dynamic mean field model

We used the model of [11] to describe the global dynamics of the whole cortex. This model binds the dynamics of *N* local nodes, composed of excitatory—inhibitory subnetworks (E—I networks), through the underlying anatomical structure which is estimated using diffusion-imaging data from healthy human subjects. The stochastic differential equations of the model describe the time evolution of the mean synaptic activity of each local node (i.e., brain region) and there are given by:

$$u_{i}{}^{\left(E\right)}=I_{0,E}+w_{EE}S_{i}{}^{\left(E\right)}+G\sum_{j}C_{ij}S_{j}^{\left(E\right)}-w_{EI,i}S_{i}{}^{\left(I\right)}+I_{ext,i},$$ (6)

$$u_{i}{}^{\left(I\right)}=I_{0,I}+w_{IE}S_{i}{}^{\left(E\right)}-w_{II}S_{i}{}^{\left(I\right)},$$ (7)

$$r_{i}{}^{\left(E\right)}=\Phi_{E}(u_{i}{}^{\left(E\right)})=\frac{a_{E}u_{i}{}^{\left(E\right)}-b_{E}}{1-\text{exp}(-d_{E}(a_{E}u_{i}{}^{\left(E\right)}-b_{E}))},$$ (8)

$$r_{i}{}^{\left(I\right)}=\Phi_{I}(u_{i}{}^{\left(I\right)})=\frac{a_{I}u_{i}{}^{\left(I\right)}-b_{I}}{1-\text{exp}(-d_{I}(a_{I}u_{i}{}^{\left(I\right)}-b_{I}))},$$ (9)

$$\frac{dS_{i}{}^{\left(E\right)}}{dt}=-\frac{S_{i}{}^{\left(E\right)}}{\tau_{E}}+(1-S_{i}{}^{\left(E\right)})\gamma r_{i}{}^{\left(E\right)}+\beta \eta_{i}^{\left(E\right)}(t),$$ (10)

$$\frac{dS_{i}{}^{\left(I\right)}}{dt}=-\frac{S_{i}{}^{\left(I\right)}}{\tau_{I}}+r_{i}{}^{\left(I\right)}+\beta \eta_{i}^{\left(I\right)}(t),$$ (11)

where $S_{i}^{E,I}$ denotes the average excitatory or inhibitory synaptic gating variable (i.e., fraction of open channels) at the local area *i* (*i* ∈ [*1*,…,*N*]). In Eqs 10 and 11 $\eta_{i}^{\left(E\right)}(t)$ and $\eta_{i}^{\left(I\right)}(t)$ are uncorrelated Gaussian noises and the noise amplitude at each node is *β* = 0.01. $r_{i}^{E,I}$ denotes the population firing rate of the excitatory (E) or inhibitory (I) population in the brain area *i*. The population firing rates are sigmoid functions (Φ_*I*_ and Φ_*E*_) of the input synaptic currents to the excitatory or inhibitory population *i* is given by $u_{i}^{E,I}$. Synaptic currents are the sum of i) local currents within the local E—I networks, ii) excitatory currents from the other local nodes, and iii) external inputs *I*_ext_. The local currents in node *i* are the sum of constants inputs to excitatory and inhibitory populations, noted *I*_0,E_ and *I*_0,I_, respectively, local excitatory-to-excitatory currents $w_{EE}S_{i}^{\left(E\right)}$, local inhibitory-to-excitatory currents $w_{EI,i}S_{i}^{\left(I\right)}$, local excitatory-to-inhibitory currents $w_{IE}S_{i}^{\left(E\right)}$, and local inhibitory-to-inhibitory currents $w_{II}S_{i}^{\left(I\right)}$. The weights of these local connections are given by: *w*_*EE*_ = 0.21; *w*_*IE*_ = 0.15; *w*_*II*_ = 1; and the feedback inhibition weight,*w*_*EI*,*i*_, is adjusted for each node *i* so that the firing rate of the local excitatory neural population is clamped around 3Hz, whenever nodes are connected or not—this regulation is known as Feedback Inhibition Control (FIC) and the algorithm to achieve it is described in [11]. It has been shown that the FIC constrain leads to a better prediction of the resting functional connectivity and a more realistic network evoked activity [11]. Local E—I networks interact through excitatory connections given by the *N*-by-*N* anatomical connectivity matrix, noted **C**. The connectivity matrix is scaled by a single global parameter, *G*, that changes the network from weakly to strongly connected and determines the dynamical state of the system. As shown in [11] the model has one single stable fixed point of low firing activity in all cortical areas, for all values of *G* within the region where the FIC regulation can be achieved. For larger values of G, long-range interactions are too strong to be compensated by FIC and the activity diverges. Finally, *I*_ext_ represents external stimulation for simulating task evoked activity: it is zero for all neural populations under resting state condition, and *I*_ext_>0 for those populations excited in the task condition.

The values of all parameters are taken from [11] and are presented in S1 Table.

### Structural connectivity matrix

Neuroanatomical structure was obtained using Diffusion Spectrum Imaging (DSI) data and tractography from five healthy right-handed male human subjects [12]. The grey matter was subdivided into 998 regions of interest (ROIs) which are grouped into 33 cortical regions per hemisphere (66 areas in total) according to anatomical landmarks (S2 Table). White matter tractography was used to estimate the fiber tract density connecting each pair of ROIs, averaged across subjects. Anatomical connectivity among the 66 cortical regions was calculated by summing all incoming fiber strengths to the corresponding ROIs of the target region, and dividing it by its region-dependent number of ROIs, resulting in a non-symmetric connectivity matrix. This normalization by the number of ROIs—which have approximately the same surface on the cortex, i.e. the same number of neurons—is required because neuronal activity is sensitive to the number of incoming fibers per neuron in the target region. As the dynamical model of one region already takes into account the effect of its internal connectivity (see below), the connection of a region to itself was set to 0 in the connectivity matrix for the simulations.

### Linear noise approximation

In the following we derive approximated equations for the statistics of the gating variables and the synaptic activity. To estimate the network’s statistics, we assume that the noise is sufficiently weak so that the state variables fluctuate around their mean value and, by linearizing the equations, we concentrate on linear fluctuations. In this way, we express the system of stochastic differential Eqs (6–11) in terms of the first- and second-order statistics of the distribution of synaptic gating variables: $\mu_{i}^{\left(m\right)}$, the expected mean gating variable of a given local neural population of type *m* (where *m = E* or *I*) of the cortical area *i*, and $P_{ij}^{\left(mn\right)}$, the covariance between gating variables of neural populations of type *m* and *n* of local cortical areas *i* and *j*, respectively. The statistics are defined as:

$$\mu_{i}^{\left(m\right)}(t)=\langle S_{i}^{\left(m\right)}(t)\rangle ,$$ (12)

$$P_{ij}^{\left(mn\right)}(t)=\langle \left[S_{i}^{\left(m\right)}(t)-\mu_{i}^{\left(m\right)}(t)\right]\left[S_{j}^{\left(n\right)}(t)-\mu_{j}^{\left(n\right)}(t)\right]\rangle ,$$ (13)

where the angular brackets <.> denote the average over realizations or “trials”. Note that, for the model, a “trial” means a realization of the system of differential Eqs (6–11). In vector form, the system of equations writes:

$$\frac{d}{dt}\left(\begin{array}{c} {\vec{S}}^{\left(E\right)}\\ {\vec{S}}^{\left(I\right)} \end{array}\right)=\left(\begin{array}{c} f^{\left(E\right)}\left({\vec{S}}^{\left(E\right)},{\vec{S}}^{\left(I\right)}\right)\\ f^{\left(I\right)}\left({\vec{S}}^{\left(E\right)},{\vec{S}}^{\left(I\right)}\right) \end{array}\right)+\vec{\eta }(t),$$ (14)

where $\vec{S}=\left\{{\vec{S}}^{\left(E\right)},{\vec{S}}^{\left(I\right)}\right\}=\left\{S_{1}^{\left(E\right)},\ldots ,S_{N}^{\left(E\right)},S_{1}^{\left(I\right)},\ldots ,S_{N}^{\left(I\right)}\right\}$, $\vec{\eta }$ is uncorrelated Gaussian noise, $f_{i}{}^{\left(E\right)}\left({\vec{S}}^{\left(E\right)},{\vec{S}}^{\left(I\right)}\right)=-\frac{S_{i}^{\left(E\right)}}{\tau_{E}}+(1-S_{i}{}^{\left(E\right)})\gamma \Phi^{\left(E\right)}(u_{i}{}^{\left(E\right)})$, and $f_{i}{}^{\left(I\right)}\left({\vec{S}}^{\left(E\right)},{\vec{S}}^{\left(I\right)}\right)=-\frac{S_{i}^{\left(I\right)}}{\tau_{I}}+\Phi^{\left(I\right)}(u_{i}{}^{\left(I\right)})$ for *i* = 1,..,*N*.

In the following we use a linear approximation of the fluctuations. As shown in [11], Taylor expanding $\vec{S}$ around $\vec{\mu }=\langle \vec{S}\rangle$, i.e. $S_{i}^{\left(m\right)}=\mu_{i}^{\left(m\right)}+\delta S_{i}^{\left(m\right)}$, up to the first order, we obtain the differential equations for the means of the gating variables and the covariance of the fluctuations around the mean. For the mean values:

$$\frac{d\mu_{i}^{\left(E\right)}}{dt}=\frac{d}{dt}\langle {\vec{S}}_{i}^{\left(E\right)}\rangle =-\frac{\mu_{i}^{\left(E\right)}}{\tau_{E}}+(1-\mu_{i}{}^{\left(E\right)})\gamma \Phi_{E}(u_{i}{}^{\left(E\right)}),$$ (15)

$$\frac{d\mu_{i}^{\left(I\right)}}{dt}=\frac{d}{dt}\langle {\vec{S}}_{i}^{\left(I\right)}\rangle =-\frac{\mu_{i}^{\left(I\right)}}{\tau_{I}}+\Phi_{I}(u_{i}{}^{\left(I\right)}),$$ (16)

where *u*_*i*_^*(m)*^ is the mean input current to the neural population *m* = *E*,*I* of cortical area *i*, defined as:

$$\vec{u}=\left(\begin{array}{c} {\vec{u}}^{\left(E\right)}\\ {\vec{u}}^{\left(I\right)} \end{array}\right)=W\vec{S}+{\vec{I}}_{0}+{\vec{I}}_{ext},$$ (17)

where **W** is a block matrix defined as:

$$W=\left[\begin{array}{cc} w_{EE}I_{N}+G.C & -D({\vec{w}}_{EI})\\ w_{IE}I_{N} & -w_{II}I_{N} \end{array}\right],$$

where **C** is the *N*x*N* anatomical matrix, *G* the global coupling parameter, **I**_*N*_ is the *N*x*N* identity matrix, $D({\vec{w}}_{EI})$ is a *N*x*N* diagonal matrix containing the weights of the feedback inhibition *w*_*EI*,*i*_ as diagonal elements, and ${\vec{I}}_{0}$ and ${\vec{I}}_{ext}$ are the vectors containing the constant and external inputs.

Let **P** being the covariance matrix between gating variables $\vec{S}$. **P** is a block matrix defined as:

$$P=\left[\begin{array}{cc} P^{\left(EE\right)} & P^{\left(EI\right)}\\ P^{\left(IE\right)} & P^{\left(II\right)} \end{array}\right].$$

The differential equation of the covariance matrix is [11]:

$$\frac{dP}{dt}=AP+PA^{T}+Q_{n},$$ (18)

where the superscript *T* is the transpose, **Q**_n_ is the covariance matrix of the noise, given by $Q_{n}=\langle \vec{\eta }(t)\vec{\eta }{\left(t\right)}^{T}\rangle$, and **A** is the Jacobian matrix given by first-order partial derivative of the nonlinear function *f* with respect to each variable *S*, evaluated at $\vec{\mu }$. **A** is a block matrix defined as:

$$A=\left[\begin{array}{cc} A^{\left(EE\right)} & A^{\left(EI\right)}\\ A^{\left(IE\right)} & A^{\left(II\right)} \end{array}\right],$$

where

$$A_{ij}^{\left(mn\right)}=\left\{\frac{\partial f_{i}{}^{\left(m\right)}(\vec{\mu })}{\partial S_{j}^{\left(n\right)}}\right\}.$$

Note that the Jacobian matrix depends on the point $\vec{\mu }$ at which it is evaluated.

The synaptic input variables $\vec{u}$ are a linear combination of the gating variables $\vec{S}$ and, thus, covariance matrix between synaptic input variables $\vec{u}$ is given by:

$$C_{v}=WPW^{T}.$$ (19)

Knowledge of the Jacobian matrix and the stationary covariance gives the stationary autocovariance of the gating variables $\vec{S}$, defined as the covariance of the process with itself at pairs of time points and given as:

$$F_{S}\left(t+\tau ,t\right)=\langle \left[\vec{S}(t+\tau )-\vec{\mu }(t+\tau )\right]{\left[\vec{S}(t)-\vec{\mu }(t)\right]}^{T}\rangle .$$ (20)

In the stationary regime **F**_***S***_(*t+τ*,*t*) depends only on *τ* and is given by:

$$F_{S}\left(\tau \right)=e^{\tau A}F_{S}(0)=e^{\tau A}P,$$ (21)

where the exponential matrix is defined as:

$$e^{\tau A}=I+\tau A+\frac{1}{2!}{\left(\tau A\right)}^{2}+\frac{1}{3!}{\left(\tau A\right)}^{3}+\ldots$$ (22)

The stationary autocovariance of the synaptic input variables $\vec{u}$ is, thus, given by:

$$F_{u}\left(\tau \right)=WF_{S}\left(\tau \right)W^{T}.$$ (23)

The autocorrelation function (ACF) of the *i*-th synaptic input variable is given by:

$${\text{ACF}}_{i}\left(\tau \right)=F_{u,i}(\tau )/F_{u,i}(0).$$ (24)

Finally, the power spectral density (PSD) of fluctuations around the fixed points is also determined by the Jacobian matrix. The cross-spectrum of the gating variables $\vec{S}$ is given as [11]:

$$\Pi_{S}(\omega )=\langle \delta \tilde{S}(\omega )\delta \tilde{S}{\left(\omega \right)}^{†}\rangle ={\left(A+i\omega \right)}^{-1}Q_{n}{\left(A^{T}-i\omega \right)}^{-1},$$ (25)

where $\delta \tilde{S}(\omega )$ is the Fourier transform of $\delta \vec{S}(t)$ and the superscript † is the conjugate transpose. The cross-spectrum of the synaptic input variables $\vec{u}$ is, thus, given by:

$$\Pi_{u}(\omega )=\langle \delta \tilde{u}(\omega )\delta \tilde{u}{\left(\omega \right)}^{†}\rangle =W\Pi_{S}(\omega )W^{†}.$$ (26)

The PSD of synaptic activity as a function of the frequency *ω* is given by the diagonal of **∏**_u_(*ω*).

Note that the different network’s statistics (variances, covariances, and PSD) are determined by the Jacobian matrix **A** that depends on the state of the nonlinear system (the elements of the **A** are derivatives evaluated at $\vec{\mu }$). Because the application of an external input changes the state of the system, therefore changing the derivatives, the network’s statistics are also changed. In other words, the nonlinear nature of the system renders the network’s statistics state-dependent.

In summary, to get the stationary network’s statistics we simulated the deterministic Eqs (15–18) and, once the stationary values of the mean synaptic gating variables ($\vec{\mu }$), the covariance matrix (**P**), and the Jacobian matrix (**A**) were reached all other statistics were computed using Eqs 19–26. All differential equations used in the present study were solved using the Euler’s method with a time step equal to d*t* = 0.1 ms. The total number of simulation steps was 10^5^, this simulation length ensures that the system reaches the stationary regime.

### Differential entropy

Once we have obtained the linear prediction of the covariance we can estimate the extent of all possible configurations of the network given by the differential entropy *H*, which expresses the entropy of a continuous variable with *n*-dimensional probability density function (p.d.f.) *f*, and writes:

$$H(f)=-\int_{D}f(\vec{x})\text{ln}f(\vec{x})d\vec{x},$$ (27)

where *D* ∈ ℝ^*n*^ is the support set of *f*, i.e., *D* = {*x*|*f*(*x*) > 0}. The entropy is related to the spread of the p.d.f., i.e., it relates to the volume occupied by a continuous random variable. The volume of the support set *D* is defined as:

$$Vol(D)=\int_{D}dx_{1}dx_{2}\ldots dx_{n}.$$ (28)

The volume of the smallest set that contains most of the p.d.f is approximately 2^*nH*(*f*)^ [48]. Thus, low entropy implies that the random variable is confined to a small effective *n*-dimensional volume and high entropy indicates that the random variable is widely dispersed.

For a *n*-dimensional normal distribution (**μ**, **∑**) with covariance matrix **∑**, the differential entropy in bits is given by the following form [48]:

$$H=\frac{1}{2}\text{ln}\left[{\left(2\pi e\right)}^{n}\text{det}\left(\Sigma \right)\right]/\text{ln}(2)=\frac{n}{2\text{ln}(2)}\left(1+\text{ln}(2\pi )\right)+\frac{1}{2\text{ln}(2)}\text{det}\left(\Sigma \right),$$ (29)

where det(Σ) is the determinant of the covariance matrix. We also calculated the differential entropy for the fMRI time-series used in [3]. For these empirical data we used two different calculations of the differential entropy. The first measure assumes that the data follows a *n*-dimensional multivariate normal distribution (*n* = 33) and is given by, first, estimating the covariance matrix of the fMRI signals for each subject (averaged across runs of the same condition, rest or task), noted $\hat{\Sigma }$, second, calculating the determinant of $\hat{\Sigma }$ as the product of the *k* non-zero singular values (*λ*) to elude singularity, and, finally, calculating the entropy as follows:

$$H=\frac{k}{2\text{ln}(2)}\left(1+\text{ln}(2\pi )\right)+\frac{1}{2\text{ln}(2)}\sum_{j=1}^{k}\text{ln}(\lambda_{j}).$$ (30)

For 16/17 subjects we found that *k* = 29 for both rest and task. For only one subject we found that *k* = *n* = 33 for both rest and task. As a second measure we used the Nilsson-Kleijn non-parametric estimator that does not assume normality and calculates the differential entropy based on nearest neighbors of a sample set [49]. Both ways of calculating the differential entropy *H* gave very similar results: the values of *H* obtained using the two methods were highly correlated (*r*_*c*_ = 0.91 for rest data and *r*_*c*_ = 0.90 for task data).

### Relative entropy

Following [50], we defined the relative entropy as the Kullback-Leibler divergence between the intrinsic noise and the synaptic activity of the network. In its general form the Kullback-Leibler divergence between two distributions *f* and *g* is defined as:

$$KLD=\int f\text{ln}\frac{f}{g}.$$ (31)

The intrinsic noise and the synaptic activity are normally distributed (see S3 Fig) and, in this case, it can be shown that the relative entropy between the intrinsic noise and the synaptic activity writes [50]:

$$KLD(\vec{u},\vec{\eta })=\frac{1}{2}\left[\text{trace}\left(Q_{n}^{-1}C_{v}\right)-\text{ln}\frac{\text{det}(C_{v})}{\text{det}(Q_{n})}-2N\right]/\text{ln}(2).$$ (32)

The relative entropy can be seen as the amount of uncertainty that is produced by the dynamical system.

## Supporting Information

S1 Text Appendix: Isolated node case. (PDF)

S1 Table Parameters of dynamic mean-field model. (DOC)

S2 Table Names and abbreviations of the brain regions considered in the human connectome from Hagmann et al. (2008) (in alphabetical order). (DOC)

S1 FigEffect of an external input on *σ*_*E*_^2^ for an isolated E-I node.
**A:** transfer functions of the E and I populations, Φ_*E*_(*u*_*E*_) and Φ_*I*_(*u*_*I*_), and their derivatives, ${\Phi^{\prime }}_{E}(u_{E})$ and ${\Phi^{\prime }}_{I}(u_{I})$. The circles indicate the corresponding values in the spontaneous condition (*I*_*ext*_ = 0). **B:** $\sigma_{E}^{2}$ as a function of *I*_*ext*_, using the solution given by the linear noise approximation (equation A19, see S1 Text) and using the approximated expression in equation A20 (see S1 Text). Parameters: *w*_*EE*_ = *w*_*IE*_ = 0.15. **C:** The spontaneous excitatory firing rate (*r*_*E0*_) is shown in color-code in the parameter space {*w*_*EE*,_ *w*_*IE*_}. For all tested couples of parameters {*w*_*EE*,_ *w*_*IE*_}, except for the parameter region delimited by the white lines, $\sigma_{E}^{2}$ is a decreasing function of *I*_ext_. The insets show the dependence of $\sigma_{E}^{2}$ on *I*_*ext*_ for two points of the parameter space (*green*: *w*_*EE*_ = 0.2, *w*_*IE*_ = 0.15; *orange*: *w*_*EE*_ = 0.6, *w*_*IE*_ = 1.15).(TIF)

S2 Fig Response of modular networks. The response of the large-scale model was examined in the case of artificial *N*-by-*N* connectivity matrices of different modularity (*N* = 66). We constructed three different random binary modular graphs **M**, defined by two parameters: the overall attachment probability or “link density”, *q*, and the proportion of links within five modules, noted *p*. The link density was fixed and equal to the one of the DTI-based matrix used in our work (*q* = 0.14), and different within-modules link probabilities (*p*) were used. The matrices can be classified as (**A**) nearly random (*p* = 0.1), (**B**) moderately clustered (*p* = 0.5), and (**C**) highly clustered (*p* = 0.9) (*top panels*). These modular matrices were scaled as *G*×*m*×**M**, where *m* is the mean value of the DTI-based matrix (*m* = 0.025) and *G* = 3.4, and integrated to the dynamic mean-field model. For each connectivity matrix **M**, the local feedback inhibition weights were regulated through FIC [11]. *Middle*: the spontaneous (rest) excitatory activity is shown in *black*; stimulus-induced (task) excitatory activity is shown in *green*. The yellow dots indicate the eight nodes receiving the external input. *Bottom*: Relation between the amount of mean synaptic change (Δm) and the amount of variance change (Δσ^2^) for the excitatory populations. We found a graded negative relation between Δm and Δσ^2^ for nearly random and moderately clustered connectivities, but this relation does not hold for highly clustered connectivities.
(TIF)

S3 Fig Distribution of synaptic activity. The joint density distribution of the simulated excitatory synaptic activity from two example model brain regions *x* and *y* is shown in color code during spontaneous (*left*) and evoked (*right*) conditions. The data was obtained by simulating the large-scale model using the system of stochastic differential Eqs (6–11), with noise intensity equal to *β* = 0.01. The individual distributions of *x* and *y* (empty bars) are excellently fitted by Gaussian distributions (solid gray lines).
(TIF)

S4 Fig Entropy and relative entropy under correlated noise. **A:** As in Fig 6, we calculated the differential entropy (*H*) and the relative entropy (KLD), but using different random non-diagonal noise covariance matrices and fixing the stimulation pattern (the brain regions receiving the external input are: r/lLOCC, r/lMT, r/lPCAL, r/lST). In this analysis, we constructed non-diagonal random noise covariance matrices by, first, generating 500 realizations of a multivariate *2N*-dimensional Gaussian process with diagonal covariance equal to **Q**_**n**_ = (*β*d*t*)^2^ **I**_*2N*_, thus obtaining 2*N* time series of 500 steps, and, second, the sample covariance of these time series was calculated and used as a random non-diagonal (due to sample errors) noise covariance. We found that the evoked synaptic activity has lower differential entropy (**B**) and lower relative entropy (**C**) than the spontaneous activity, for both excitatory (*blue*) and inhibitory (*green*) synaptic activity. Parameters: *N* = 66, *β* = 0.01, d*t* = 0.1 ms.
(TIF)

S1 Dataset BOLD fMRI data (TR 2.16 s) acquired in 17 subjects. Each subject completed 8 fMRI runs (4 rest runs and 4 task runs). Each run has 194 frames. The ASCII file *Rest_fMRI* contains *T* rows corresponding to all frames for all runs and all subjects in the rest condition (*T* = 194×4×17). It has *N*+1 columns: columns 1–*N* correspond to the BOLD activity for each of the *N* ROIs and the last column indicates the subject number (*N* = 33). The ASCII file *Task_fMRI* contains *T* rows corresponding to all frames for all runs and all subjects in the task condition (*T* = 194×4×17). It has *N*+2 columns: columns 1–*N* correspond to the BOLD activity for each of the *N* ROIs, column *N*+1 indicates the subject number, and a value 1 in column *N*+2 indicates the onset of the stimulus. For each run the first 4 volumes were removed. The labels of the brain regions are contained in file *33ROIs_labels*.*txt*.
(ZIP)

S2 Dataset The ASCII file *Struct_Conn* contains the 66-by-66 structural connectivity matrix, obtained using Diffusion Spectrum Imaging (DSI) data and tractography from five healthy right-handed male human subjects [12]. The labels of the brain regions are contained in file *66ROIs_labels*.*txt*.
(ZIP)
