# Supplementary material for: Task-Driven Activity Reduces the Cortical Activity Space of the Brain: Experiment and Whole-Brain Modeling
Source: PLoS Comput Biol. 2015 Aug 28;11(8):e1004445. doi: 10.1371/journal.pcbi.1004445 (PMC4552873; doi:10.1371/journal.pcbi.1004445)
Supplement: S1 Text — (PDF) [file pcbi.1004445.s001.pdf]

Appendix: Isolated node case

For an isolated E-I node, using the linear noise approximation, it is possible to explicitly solve the equation of the stationary covariance (equation 18) and to isolate the dominant factors that affect the variance of the excitatory synaptic activity, i.e., σ_E^2=Var[u_E], when an external input is imposed. The evolution equations for the mean values of the gating variables are:

$$\frac{d\mu_E}{dt} = \frac{d}{dt}\langle S_E \rangle = -\frac{\mu_E}{\tau_E} + (1-\mu_E)\Phi_E(u_E), \tag{A1}$$

$$\frac{d\mu_I}{dt} = \frac{d}{dt}\langle S_I \rangle = -\frac{\mu_I}{\tau_I} + \Phi_I(u_I), \tag{A2}$$

$$u_E = w_{EE}\mu_E - w_{EI}\mu_I + I_{0,E} + I_{ext}, \tag{A3}$$

$$u_I = w_{IE}\mu_E - w_{II}\mu_I + I_{0,I}, \tag{A4}$$

where $w_{EE} = 0.21$, $w_{IE} = 0.15$, $w_{II} = w_{EI} = 1$, $\tau_E = 100\text{ms}$, $\tau_I = 10\text{ms}$, and the transfer functions, $\Phi_E(u_E)$ and $\Phi_I(u_I)$, and their derivatives, $\Phi'_E(u_E)$ and $\Phi'_I(u_I)$, are shown in **S1 Fig. A**. Note that Φ'_I is much higher than Φ_E, Φ_I, and Φ'_E.

We studied the fluctuations around the stationary state $\boldsymbol{\mu}^* = \left(\mu_E^*, \mu_I^*\right)$, for which we have:

$$0 = -\frac{\mu_E^*}{\tau_E} + (1-\mu_E^*)\Phi_E(u_E^*), \tag{A5}$$

$$0 = -\frac{\mu_I^*}{\tau_I} + \Phi_I(u_I^*), \tag{A6}$$

where $\mathbf{u}^* = \left(u_E^*, u_I^*\right)$ is the stationary mean synaptic activity, given by: $u_E^* = w_{EE}\mu_E^* - \mu_I^* + I_{0,E} + I_{ext}$ and $u_I^* = w_{IE}\mu_E^* - \mu_I^* + I_{0,I}$.

In the stationary regime, the covariance matrix of the fluctuations of the synaptic gating variables around the stationary state $\boldsymbol{\mu}^* = \left(\mu_E^*, \mu_I^*\right)$ is given by:

$$\mathbf{AP} + \mathbf{PA}^{\mathbf{T}} + \mathbf{Q_n} = \mathbf{0}. \tag{A7}$$

In equation (A7), $\mathbf{P} = \begin{pmatrix} \rho_{EE} & \rho_{EI} \\ \rho_{EI} & \rho_{II} \end{pmatrix}$ is the covariance of gating variables' fluctuations, where $\rho_{EE} = \left\langle \left(S_E - \mu_E^*\right)^2 \right\rangle$, $\rho_{II} = \left\langle \left(S_I - \mu_I^*\right)^2 \right\rangle$ and $\rho_{EI} = \left\langle \left(S_E - \mu_E^*\right)\left(S_I - \mu_I^*\right) \right\rangle$; $\mathbf{Q_n}$ is the noise covariance matrix [for uncorrelated noise $\mathbf{Q_n}$ is diagonal: $\mathbf{Q_n} = \begin{pmatrix} q & 0 \\ 0 & q \end{pmatrix}$]; $\mathbf{A} = \begin{pmatrix} A_{EE} & A_{EI} \\ A_{EI} & A_{II} \end{pmatrix}$ is the Jacobian matrix evaluated at $\boldsymbol{\mu}^*$.

Note that the Jacobian matrix depends on the point $\boldsymbol{\mu}^*$ at which it is evaluated, i.e., it

depends on the state of the nonlinear system. Because the application of an external input changes the state of the system, the network's covariance is also changed. In other words, the nonlinear nature of the system renders the network's statistics state-dependent.

The elements of the Jacobian matrix are given by:

$$A_{EE}\left(u_E^*\right) = -\frac{1}{\tau_E} + \frac{w_{EE}\Phi_E'\left(u_E^*\right)}{1+\tau_E\Phi_E\left(u_E^*\right)} - \Phi_E\left(u_E^*\right), \tag{A8}$$

$$A_{EI}\left(u_E^*\right) = -\frac{\Phi_E'\left(u_E^*\right)}{1+\tau_E\Phi_E\left(u_E^*\right)}, \tag{A9}$$

$$A_{IE}\left(u_I^*\right) = w_{IE}\Phi_I'\left(u_I^*\right), \tag{A10}$$

$$A_{II}\left(u_I^*\right) = -\frac{1}{\tau_I} - \Phi_I'\left(u_I^*\right). \tag{A11}$$

Solving equation (A7), we get:

$$\rho_{EE} = -\frac{q + 2A_{EI}\rho_{EI}}{2A_{EE}}, \tag{A12}$$

$$\rho_{II} = -\frac{q + 2A_{IE}\rho_{EI}}{2A_{II}}, \tag{A13}$$

$$\rho_{EI} = -\frac{A_{IE}\rho_{EE} + A_{EI}\rho_{II}}{A_{EE} + A_{II}}. \tag{A14}$$

Given equations A3 and A4, the variance of the fluctuations of the excitatory synaptic activity around is given by:

$$\sigma_E^2 = \left\langle \left(u_E - u_E^*\right)^2 \right\rangle = w_{EE}^2\rho_{EE} + \rho_{II} - 2w_{EE}\rho_{EI}. \tag{A15}$$

The set of equations A12–15 give all the information to express σ_E^2 as a function of $\mathbf{u}^*$. Inserting equations (A12) and (A13) into (A14) we get:

$$\rho_{EE} = -\frac{q}{2}\frac{A_{II}\left[A_{II} + A_{EE}\right] - A_{EI}A_{IE} + A_{EI}^2}{\left[A_{II} + A_{EE}\right]\left[A_{EE}A_{II} - A_{EI}A_{IE}\right]}, \tag{A16}$$

$$\rho_{II} = -\frac{q}{2}\frac{A_{EE}\left[A_{II} + A_{EE}\right] - A_{EI}A_{IE} + A_{IE}^2}{\left[A_{II} + A_{EE}\right]\left[A_{EE}A_{II} - A_{EI}A_{IE}\right]}, \tag{A17}$$

$$\rho_{EI} = \frac{q}{2}\frac{A_{EI}A_{EE} + A_{IE}A_{II}}{\left[A_{II} + A_{EE}\right]\left[A_{EE}A_{II} - A_{EI}A_{IE}\right]}. \tag{A18}$$

Thus, σ_E^2 can be written as:

$$\sigma_E^2 = -\frac{q}{2\left(A_{II}+A_{EE}\right)}\left[w_{EE}^2+1+\frac{U}{Z}\right], \tag{A19}$$

where:

$$Z = A_{EE}A_{II} - A_{EI}A_{IE} = \left(\frac{1}{\tau_I}+\Phi'_I\right)\left(\frac{1}{\tau_E}+\Phi_E\right)+\left(w_{IE}-w_{EE}\right)\frac{\Phi'_E\Phi'_I}{1+\tau_E\Phi_E}-\frac{w_{EE}}{\tau_I}\frac{\Phi'_E}{1+\tau_E\Phi_E},$$

$$U = A_{II}^2\left(w_{EE}-w_{IE}\right)^2+\left(\frac{1}{\tau_E}+\Phi_E\right)^2+\frac{2}{\tau_I}A_{II}\left(w_{EE}^2-w_{EE}w_{IE}\right)+\frac{w_{IE}^2}{\tau_I^2},$$

and where we have used the simplified notation: $\Phi_E = \Phi_E\left(u_E^*\right)$, $\Phi_I = \Phi_I\left(u_I^*\right)$, $\Phi'_E = \Phi'_E\left(u_E^*\right)$ and $\Phi'_I = \Phi'_I\left(u_I^*\right)$.

To get some intuition on the main factors governing the dependence of σ_E^2 on I_{ext}, we studied the simple case $w_{EE} = w_{IE} = w$, for which some terms in U and Z vanish. Since: $\frac{1}{\tau_I}+\Phi'_I+\frac{1}{\tau_E}+\Phi_E \gg \frac{w\Phi'_E}{1+\tau_E\Phi_E}$, σ_E^2 can be approximated as:

$$\sigma_E^2 \approx \frac{q}{2\left(\frac{1}{\tau_I}+\Phi'_I+\frac{1}{\tau_E}+\Phi_E\right)}\left[w_{EE}^2+1+\frac{\left(w/\tau_I\right)^2+\left(\frac{1}{\tau_E}+\Phi_E\right)^2}{\left(\frac{1}{\tau_I}+\Phi'_I\right)\left(\frac{1}{\tau_E}+\Phi_E\right)}\right]. \tag{A20}$$

As shown in **S1 Fig. B** the above expression gives a good approximation of σ_E^2.

Thus, because $\Phi_E(u_E)$ and $\Phi'_I(u_I)$ are increasing functions, σ_E^2 is a decreasing function of $\mathbf{u}^*$. Hence, the decrease of the variance of the excitatory synaptic activity as a function of the external input is a consequence of the nonlinearity of the transfer functions. Note that the transfer functions become linear for a sufficiently strong I_{ext}, in this regime the system is nearly linear and, thus, the variance becomes independent of the input (**S1 Fig. B**). However, it has been shown that real neurons operate in the nonlinear regime (Priebe et al., 2004; Priebe and Ferster, 2005) and, thus, variance saturation might not be observed empirically.

We further examined the dependence of σ_E^2 on I_{ext} in the parameter space $\{w_{EE}, w_{IE}\}$ (**S1 Fig. C**). The color code in **S1 Fig. C** shows the spontaneous excitatory firing rate (r_{E0}). As long as w_{EE} is not too high compared to w_{IE}, the excitatory firing rate at the spontaneous state is

realistic (<10 spikes/s). We found that σ_E^2 is a decreasing function of I_{ext} for all tested couples of parameters $\{w_{EE}, w_{IE}\}$ except for the parameter region delimited by the white lines, for which σ_E^2 presents a maximum for $I_{\text{ext}}>0$. Thus, depending on the connectivity parameters, the spontaneous state of low activity can present two different behaviors of $\sigma_E^2(I_{\text{ext}}=0)$ that can be maximal or not.

In conclusion, the decrease of synaptic activity's variance in response to an external input is determined by nonlinearities and connectivity.

References:

Priebe, N., Mechler, F., Carandini, M., & Ferster, D. (2004). The contribution of spike threshold to the dichotomy of cortical simple and complex cells. Nat. Neurosci., 7 , 1113-22.

Priebe, N. J., & Ferster, D. (2005). Direction selectivity of excitation and inhibition in simple cells of the cat primary visual cortex. Neuron, 45 , 133-45.
