# Supplementary material for: Task-Driven Activity Reduces the Cortical Activity Space of the Brain: Experiment and Whole-Brain Modeling
Source: PLoS Comput Biol. 2015 Aug 28;11(8):e1004445. doi: 10.1371/journal.pcbi.1004445 (PMC4552873; doi:10.1371/journal.pcbi.1004445)
Supplement: S1 Table — (DOC) [file pcbi.1004445.s002.doc]

| Excitatory gating variables |
| --- |
| $a_{E}$= 310 (nC^-1^) |
| $b_{E}$=125 (Hz) |
| $d_{E}$=0.16 (s) |
| $\tau_{E}=\tau_{NMDA}$=100 (ms) |
| *I_0,E_=*0.3820 (nA) |
| Inhibitory gating variables |
| $a_{I}$= 615 (nC^-1^) |
| $b_{I}$=177 (Hz) |
| $d_{I}$=0.087 (s) |
| $\tau_{I}=\tau_{GABA}$= 10 (ms) |
| *I_0,I_ =*0.2674 (nA) |
| Fixed local connectivity parameters |
| *w_E E_=*0.21 |
| *w_II_ =*1 |
| *W_IE_ =*0.15 |
| kinetic parameter |
| $\gamma$=0.641/1000 |
