# Supplementary material for: Task-Driven Activity Reduces the Cortical Activity Space of the Brain: Experiment and Whole-Brain Modeling
Source: PLoS Comput Biol. 2015 Aug 28;11(8):e1004445. doi: 10.1371/journal.pcbi.1004445 (PMC4552873; doi:10.1371/journal.pcbi.1004445)
Supplement: S2 Table — (DOC) [file pcbi.1004445.s003.doc]

| Brain region | Abbreviation |
| --- | --- |
| **Bank of the superior temporal sulcus** | **BSTS** |
| **Caudal anterior cingulate cortex** | **CAC** |
| **Caudal middle frontal cortex** | **CMF** |
| **Cuneus** | **CUN** |
| **Entorhinal cortex** | **ENT** |
| **Frontal pole** | **FP** |
| **Fusiform gyrus** | **FUS** |
| **Inferior parietal cortex** | **IP** |
| **Isthmus of the cingulate cortex** | **ISTC** |
| **Inferior temporal cortex** | **IT** |
| **Lingual gyrus** | **LING** |
| **Lateral occipital cortex** | **LOCC** |
| **Lateral orbitofrontal cortex** | **LOF** |
| **Medial orbitofrontal cortex** | **MOF** |
| **Middle temporal cortex** | **MT** |
| **Paracentral lobule** | **PARC** |
| **Parahippocampal cortex** | **PARH** |
| **Posterior cingulate cortex** | **PC** |
| **Pericalcarine cortex** | **PCAL** |
| **Precuneus** | **PCUN** |
| **Pars opercularis** | **POPE** |
| **Pars orbitalis** | **PORB** |
| **Precentralgyrus** | **PREC** |
| **Postcentralgyrus** | **PSTC** |
| **Pars triangularis** | **PTRI** |
| **Rostral anterior cingulate cortex** | **RAC** |
| **Rostral middle frontal cortex** | **RMF** |
| **Superior frontal cortex** | **SF** |
| **Supramarginalgyrus** | **SMAR** |
| **Superior parietal cortex** | **SP** |
| **Superior temporal cortex** | **ST** |
| **Temporal pole** | **TP** |
| **Transverse temporal cortex.** | **TT** |
