# Supplementary figures and images for: Task-Driven Activity Reduces the Cortical Activity Space of the Brain: Experiment and Whole-Brain Modeling
Source: PLoS Comput Biol. 2015 Aug 28;11(8):e1004445. doi: 10.1371/journal.pcbi.1004445 (PMC4552873; doi:10.1371/journal.pcbi.1004445)

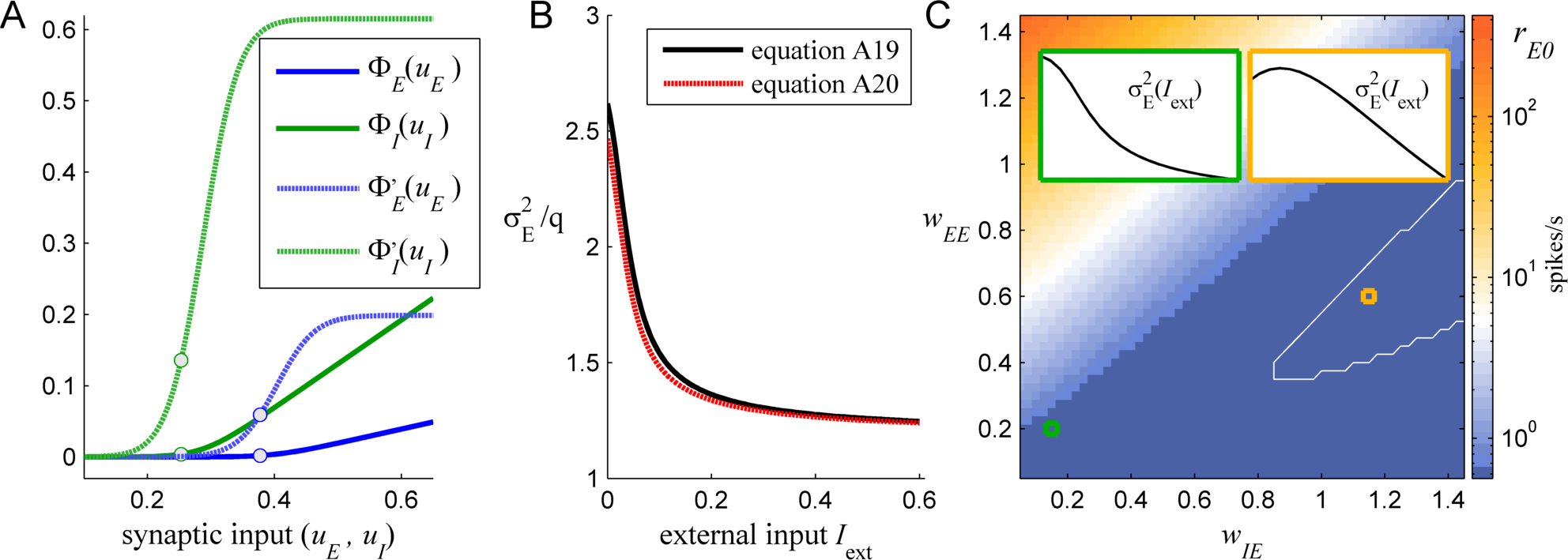

Supplement: S1 Fig — A: transfer functions of the E and I populations, ΦE(u E) and ΦI(u I), and their derivatives, Φ′E(uE) and Φ′I(uI). The circles indicate the corresponding values in the spontaneous condition (I ext = 0). B: σE2 as a function of I ext, using the solution given by the linear noise approximation (equation A19, see S1 Text) and using the approximated expression in equation A20 (see S1 Text). Parameters: w EE = w IE = 0.15. C: The spontaneous excitatory firing rate (r E0) is shown in color-code in the parameter space {w EE, w IE}. For all tested couples of parameters {w EE, w IE}, except for the parameter region delimited by the white lines, σE2 is a decreasing function of I ext. The insets show the dependence of σE2 on I ext for two points of the parameter space (green: w EE = 0.2, w IE = 0.15; orange: w EE = 0.6, w IE = 1.15). (TIF) [file pcbi.1004445.s004.tif]

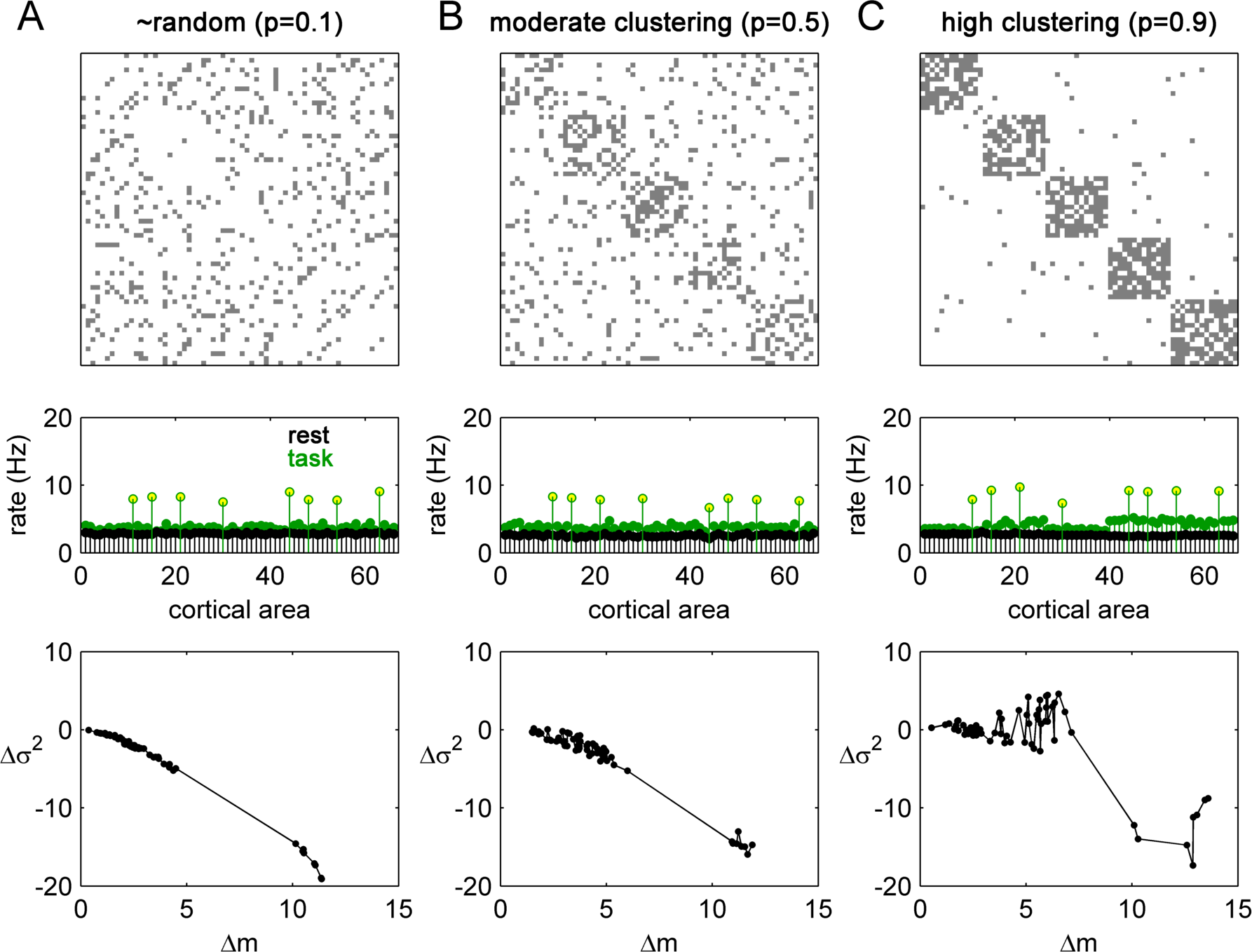

Supplement: S2 Fig — The response of the large-scale model was examined in the case of artificial N-by-N connectivity matrices of different modularity (N = 66). We constructed three different random binary modular graphs M, defined by two parameters: the overall attachment probability or “link density”, q, and the proportion of links within five modules, noted p. The link density was fixed and equal to the one of the DTI-based matrix used in our work (q = 0.14), and different within-modules link probabilities (p) were used. The matrices can be classified as (A) nearly random (p = 0.1), (B) moderately clustered (p = 0.5), and (C) highly clustered (p = 0.9) (top panels). These modular matrices were scaled as G×m×M, where m is the mean value of the DTI-based matrix (m = 0.025) and G = 3.4, and integrated to the dynamic mean-field model. For each connectivity matrix M, the local feedback inhibition weights were regulated through FIC [11]. Middle: the spontaneous (rest) excitatory activity is shown in black; stimulus-induced (task) excitatory activity is shown in green. The yellow dots indicate the eight nodes receiving the external input. Bottom: Relation between the amount of mean synaptic change (Δm) and the amount of variance change (Δσ2) for the excitatory populations. We found a graded negative relation between Δm and Δσ2 for nearly random and moderately clustered connectivities, but this relation does not hold for highly clustered connectivities. (TIF) [file pcbi.1004445.s005.tif]

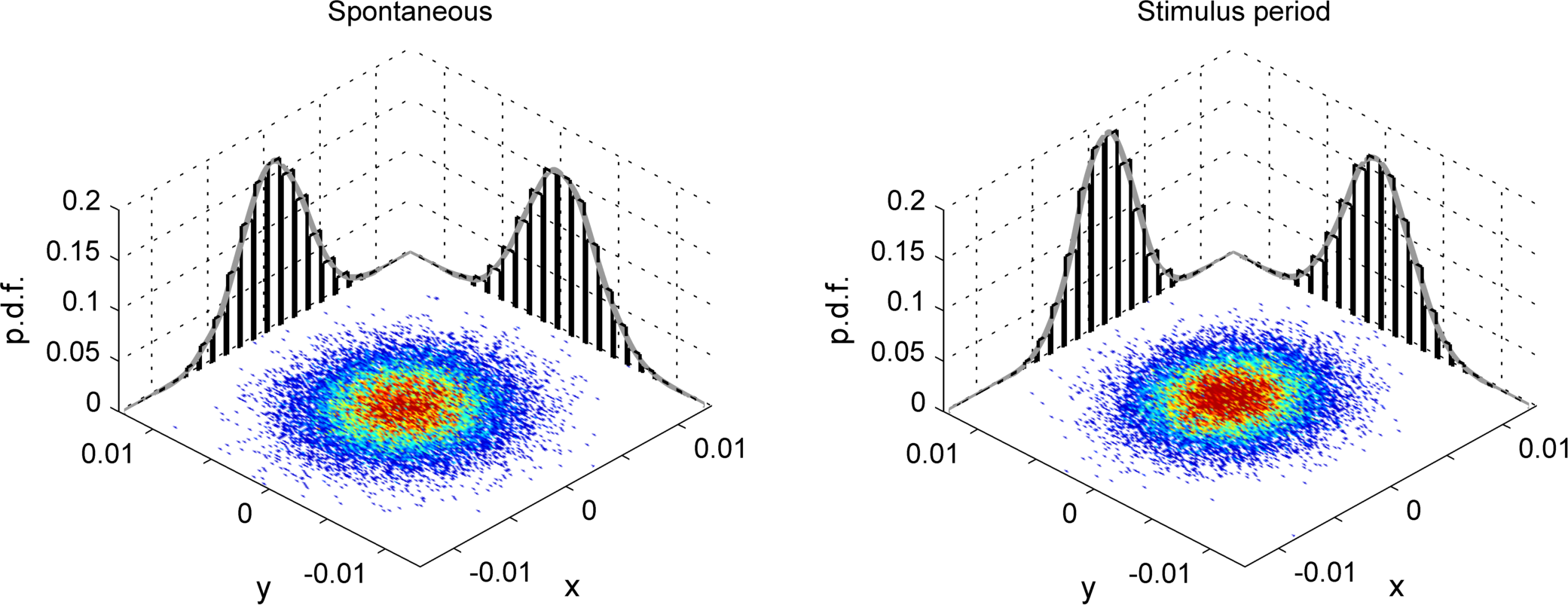

Supplement: S3 Fig — The joint density distribution of the simulated excitatory synaptic activity from two example model brain regions x and y is shown in color code during spontaneous (left) and evoked (right) conditions. The data was obtained by simulating the large-scale model using the system of stochastic differential Eqs (6–11), with noise intensity equal to β = 0.01. The individual distributions of x and y (empty bars) are excellently fitted by Gaussian distributions (solid gray lines). (TIF) [file pcbi.1004445.s006.tif]

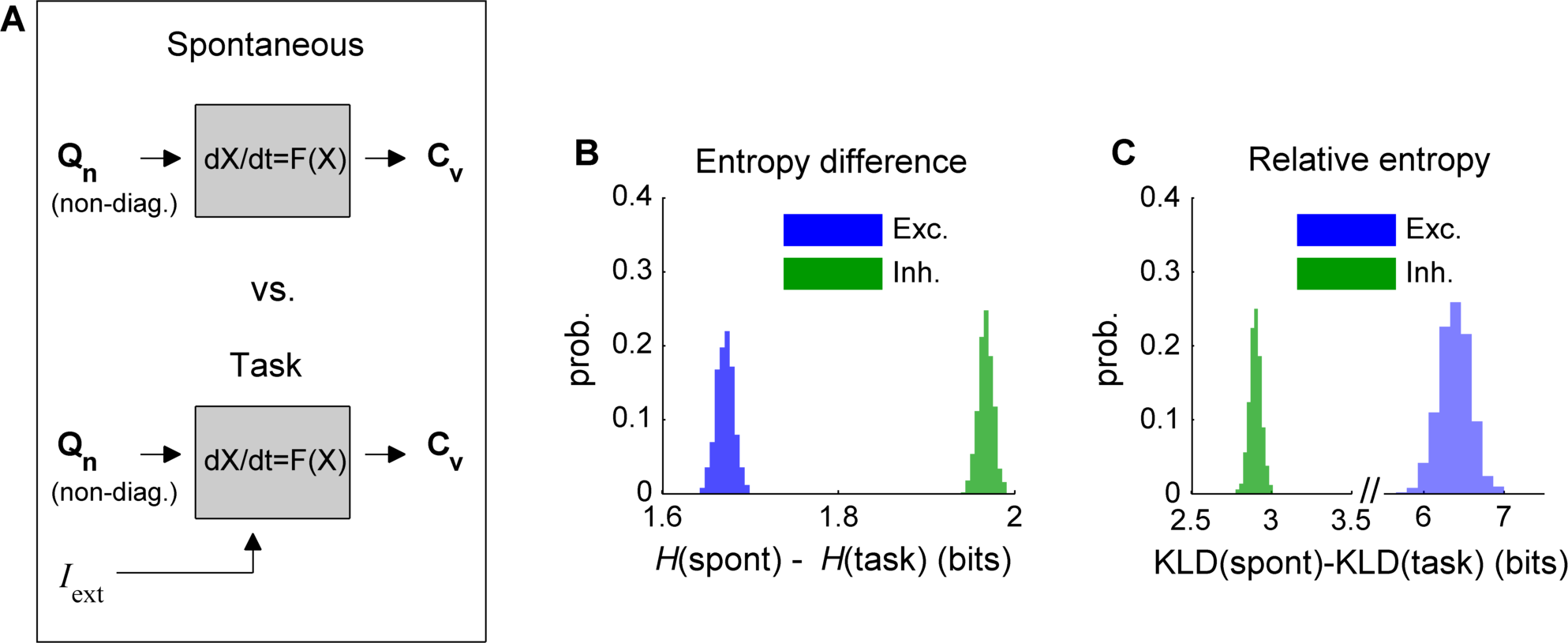

Supplement: S4 Fig — A: As in Fig 6, we calculated the differential entropy (H) and the relative entropy (KLD), but using different random non-diagonal noise covariance matrices and fixing the stimulation pattern (the brain regions receiving the external input are: r/lLOCC, r/lMT, r/lPCAL, r/lST). In this analysis, we constructed non-diagonal random noise covariance matrices by, first, generating 500 realizations of a multivariate 2N-dimensional Gaussian process with diagonal covariance equal to Q n = (βdt)2 I 2N, thus obtaining 2N time series of 500 steps, and, second, the sample covariance of these time series was calculated and used as a random non-diagonal (due to sample errors) noise covariance. We found that the evoked synaptic activity has lower differential entropy (B) and lower relative entropy (C) than the spontaneous activity, for both excitatory (blue) and inhibitory (green) synaptic activity. Parameters: N = 66, β = 0.01, dt = 0.1 ms. (TIF) [file pcbi.1004445.s007.tif]
